# Structural and Thermodynamic Discrimination between Agonists and Antagonists of Retinoic Acid Receptor γ and the Vitamin D Receptor

**DOI:** 10.1021/acs.jcim.6c00912

**Published:** 2026-06-30

**Authors:** Teresa Żołek, Szymon Sutuła, Andrzej Kutner

**Affiliations:** 1 Department of Organic and Physical Chemistry, Faculty of Pharmacy, 37803 Medical University of Warsaw, 1 Banacha, Warsaw 02-097, Poland; 2 Cryomicroscopy and Electron Diffraction Core Facility, Centre of New Technologies, 49605 University of Warsaw, 2c Banacha, Warsaw 02-097, Poland; 3 Department of Drug Chemistry, Pharmaceutical and Biomedical Analysis, Faculty of Pharmacy, 37803 Medical University of Warsaw, 1 Banacha, Warsaw 02-097, Poland

## Abstract

Synthetic ligands of retinoic acid receptor γ (RARγ) and vitamin D receptor (VDR) can act as agonists or antagonists by reshaping receptor conformation and cofactor recruitment, yet the structural and energetic determinants of this selectivity remain incompletely defined. Here, we present an integrated structural and thermodynamic analysis combining conformational characterization, molecular dynamics simulations, and molecular mechanics Poisson–Boltzmann surface area calculations to compare representative agonists and antagonists of human RARγ and VDR. Binding free energies and effective enthalpic contributions were evaluated for selected ligands, whereas the apparent entropic term, estimated indirectly as −*T*Δ*S*_app_ = Δ*G*_bind_ – Δ*H*_eff_, was used only as a qualitative internal descriptor. The thermodynamic profiles indicate that effective enthalpic stabilization is a more informative comparative descriptor than binding free energy alone, with clear agonist–antagonist separation for RARγ and a directional, less statistically resolved trend for VDR. These energetic patterns were interpreted alongside ligand-dependent stabilization of helix 12 and receptor–ligand interaction networks. Newly determined MicroED structures of AGN194310 and AGN205728 were incorporated as ligand-specific conformational references, improving structural definition without being treated as direct evidence of receptor-bound bioactive conformations. Comparative analysis of RARγ and VDR suggests that functional selectivity is not governed solely by affinity but emerges from the interplay among ligand-binding energetics, interaction networks, and receptor conformational dynamics. These results provide a retrospective mechanistic benchmark for interpreting agonist- and antagonist-associated behavior in nuclear receptors and may guide future studies on functionally selective modulators.

## Graphical abstract

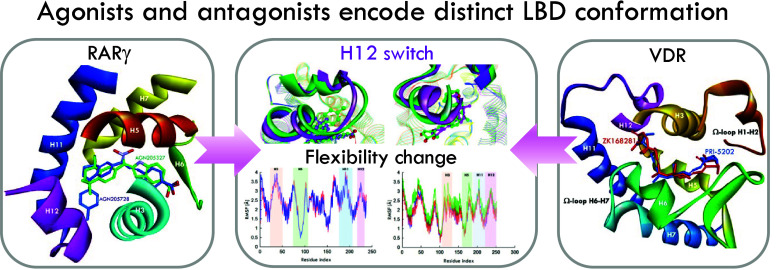

## 1 Introduction

Nuclear receptors (NRs) constitute a major class of ligand-regulated transcription factors that control metabolic, developmental, and homeostatic pathways.^1^ Their functional states are governed not only by ligand occupancy but also by ligand-induced remodeling of conformational ensembles within the ligand-binding domain (LBD). In NRs, ligand efficacy is closely coupled to the conformational organization of the LBD, particularly the position and stability of helix 12 (H12). H12 contributes directly to the activation function-2 (AF-2)/coregulator-binding surface and acts as a ligand-sensitive structural element involved in distinguishing agonist-, antagonist-, and partial agonist-like receptor states. In agonist-compatible conformations, H12 adopts an orientation that supports formation of the coactivator-binding groove, whereas antagonist binding may destabilize, displace, or reorient H12, thereby impairing productive AF-2 organization and coregulator recruitment.^2–4^ NR-specific examples support this conformational interpretation of ligand efficacy. In androgen receptor (AR) models, agonist binding promotes an H12 arrangement that closes over the ligand-binding pocket and contributes to the formation of the coactivator-binding cleft, whereas antagonist binding can force H12 into an alternative orientation incompatible with the active AF-2 surface.^5^ Similar H12-centered mechanisms have been described for vitamin D receptor (VDR) antagonists, including ZK168281, which prevents optimal folding of H12 and thereby interferes with coactivator recruitment.^6^ Studies of peroxisome proliferator-activated receptor γ (PPARγ) partial agonists indicate that ligand efficacy is not always governed by H12 stabilization alone but may also involve distributed stabilization of additional LBD regions.^7^ These examples illustrate that agonism and antagonism in NRs are better understood as ligand-dependent shifts in conformational ensembles, H12 positioning, and AF-2-associated allosteric coupling than as direct consequences of binding affinity alone. This conformational framework is directly relevant to retinoic acid receptor γ (RARγ) and VDR, the two NRs analyzed in this study. In RARγ, ligand-induced positioning of H12 is closely linked to formation or disruption of an activation-compatible AF-2 surface, providing a structural basis for distinguishing agonist- and antagonist-like receptor states.^8,9^ Similarly, in VDR, the functional response to agonists, partial agonists, and antagonists is associated with ligand-dependent organization of the ligand-binding pocket, the C-terminal activation H12, and the AF-2 region.^6,10^ Thus, in both receptors, ligand efficacy cannot be inferred from binding affinity alone but depends on how ligand binding reshapes local receptor geometry, specifically H12 positioning, and AF-2 domain stabilization.

Molecular dynamics (MD) simulations provide a powerful framework for examining these ligand-dependent conformational effects, but their interpretation depends on the sampling protocol and accessible simulation time scales. Previous MD studies of NR LBDs have used a range of strategies, from conventional nanosecond-scale simulations aimed at comparing local flexibility and interaction patterns to enhanced sampling approaches designed to probe larger H12 transitions and rare conformational exchange events.^11–13^ Complementary analyses, including dynamic cross-correlation matrices, principal component analysis, free-energy landscapes, and structural community approaches, have also been employed to characterize ligand-dependent allosteric coupling among the NR LBD, the H12/AF-2 region, and coregulator recognition surfaces.^14^ For example, solute-tempering simulations of the VDR LBD have shown that enhanced sampling can reveal ligand-dependent H12 responses that may be difficult to observe using conventional MD alone.^13^ This distinction is particularly important when interpreting results obtained from standard MD protocols. Such simulations can provide robust and reproducible information on local stability, interaction persistence, and region-specific flexibility, but they should not be assumed to exhaustively sample all relevant large-scale H12 rearrangements. Therefore, in the present study, multiple independent MD replicas are used as a comparative, rather than exhaustive, sampling strategy to evaluate ligand-dependent local dynamical signatures under identical simulation conditions.

Thermodynamic analysis has also been applied to NR systems, although less often as an explicitly integrated descriptor of the ligand functional class. Experimental thermodynamic studies of steroid and other NRs have examined ligand binding to estrogen, progesterone, and androgen receptors, illustrating that affinity alone does not fully describe the energetic basis of receptor–ligand recognition.^3,15,16^ Thermodynamic characterization of the constitutive androstane receptor (CAR) further showed that agonist binding can modulate coactivator affinity through coupled enthalpic and entropic contributions.^17^ In VDR, computational analyses of agonist and antagonist binding have linked ligand-dependent receptor states to shifts in conformational and interaction equilibria and downstream functional outcomes.^18^ Collectively, these studies support the relevance of thermodynamic descriptors for NR function but also indicate that such quantities should be interpreted together with structural and dynamical information rather than as isolated predictors of ligand efficacy.

In the context of cancer-related NR signaling, RARγ and VDR provide a useful paired comparative model for examining structurally distinct mechanisms of functional modulation. Although these receptors regulate different transcriptional programs, pharmacological inhibition of RARγ and activation of VDR have been investigated as complementary strategies that may convergently promote attenuation of malignant proliferation in selected oncogenic contexts. RARγ signaling has been associated with the maintenance of proliferative, stem-like, or therapy-resistant phenotypes in specific cancer models, whereas pharmacological inhibition of RARγ has been reported to reduce colony formation and promote growth arrest or cell death in selected cancer cell systems.^15,19^ In contrast, activation of VDR by 1,25-dihydroxyvitamin D_3_ (1,25D3) and selected vitamin D analogues has been associated with antiproliferative, prodifferentiation, and proapoptotic responses in multiple cancer models.^20^ However, therapeutic exploitation of VDR agonism is limited by calcemic liability, which has motivated the development of low-calcemic or tissue-selective VDR ligands.^19^ Thus, although RARγ and VDR regulate distinct transcriptional programs, RARγ antagonism and VDR agonism together provide a complementary framework for investigating how ligand-dependent thermodynamic and conformational features give rise to functionally selective NR modulation. This paired-receptor framework, together with the key structural features analyzed in this work, is summarized in Figure 1.

**Figure 1:**
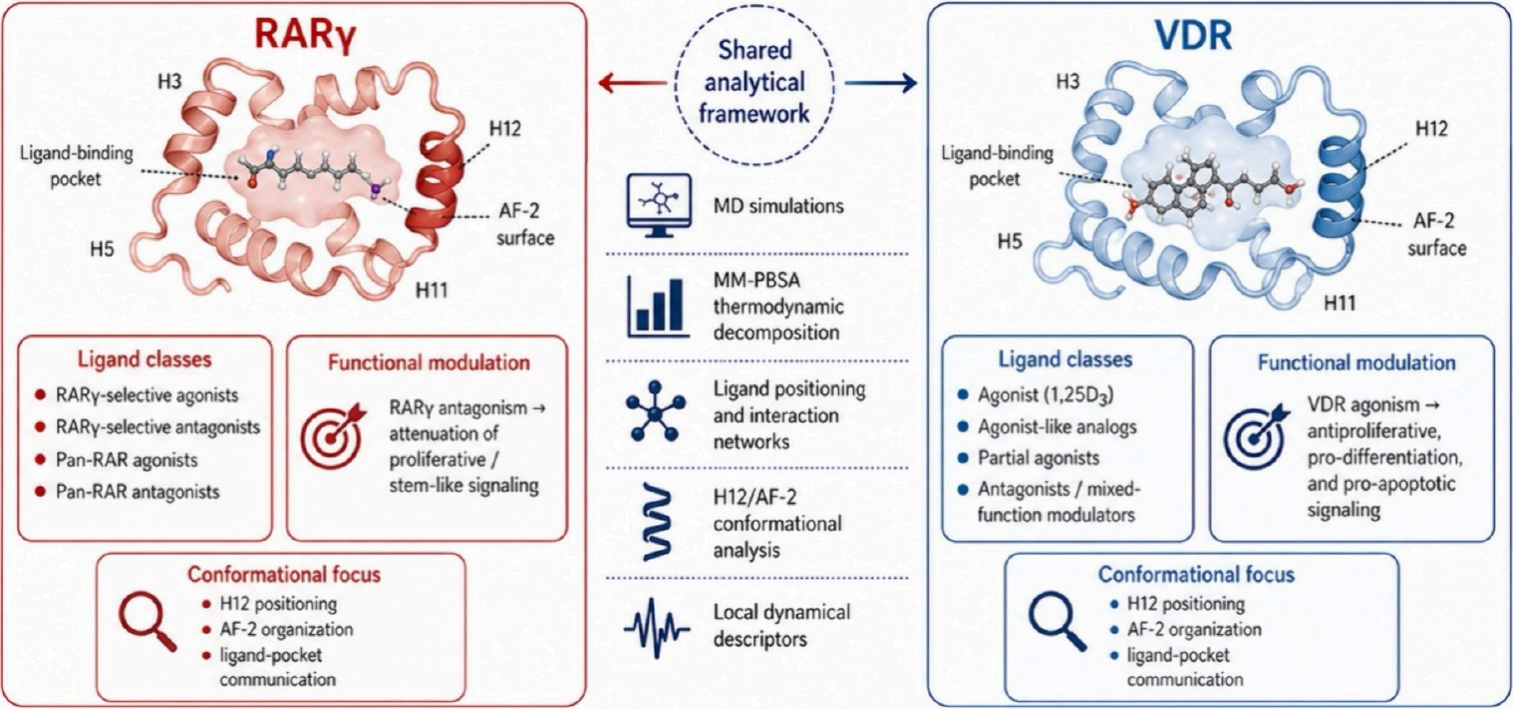
Conceptual overview of the paired RARγ-VDR framework explored in this study, highlighting both conserved and receptor-specific LBD architecture, ligand-dependent H12/AF-2 regulation, distinct ligand-binding pocket organization, and their corresponding complementary pharmacological strategies.

Despite substantial development of RARγ and VDR ligands, achieving robust functional selectivity remains challenging. Potent and subtype-selective RARγ antagonists remain relatively scarce, whereas VDR agonists with potential anticancer activity are often constrained by dose-limiting calcemic side effects. These challenges underscore the need for computational strategies that go beyond binding affinity alone to evaluate how ligand binding reshapes receptor conformational states, thermodynamic profiles, and AF-2-associated regulatory determinants. The RARγ ligand subset was curated to include chemically and functionally representative agonists, subtype-selective antagonists, and pan-RAR compounds. In this context, pan-RAR activity refers to activity across RARα, RARβ, and RARγ subtypes. The chemical structures of the *h*RARγ ligands are shown in Scheme 1, and their reported functional or binding activities, metric type, subtype selectivity, and functional annotation are summarized in Table 1.

**Scheme 1:**
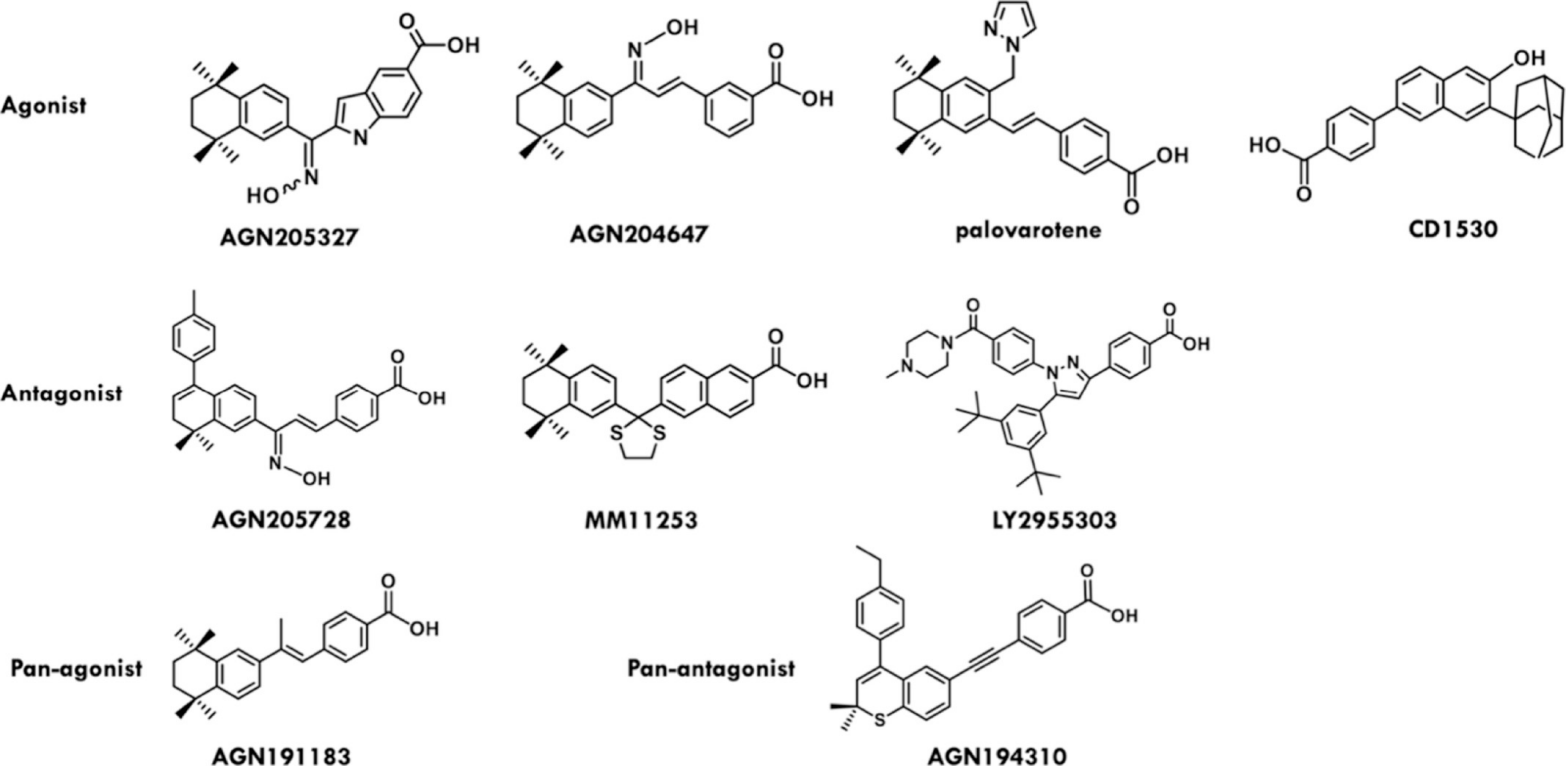
Structures of RARγ Ligands Investigated in This Study.

**Table 1:** Reported In Vitro Activity or Binding Data for the RARγ Ligands Included in this Study.^a^

compound	functional annotation	reported in vitro values (nM)	metric type	selectivity/target profile	comments
CD1530	agonist	1.8	AC_50_	RARγ-preferential	most potent agonist in this set in vitro^21^
palovarotene	agonist	n.r.	EC_50_	RARγ-selective	clinically validated RARγ agonist; reported functional activity, depends on assay context (FDA NDA 215559)
AGN205327	agonist	32.0	EC_50_	RARγ-selective	moderate potency compared with CD1530 ^22^
AGN204647	agonist	n.d.	n.r.	RARγ-selective	described in patent sources (US10213401B2/WO2017214575); directly comparable EC_50_/*K*_i_ values were not available
AGN191183	agonist	∼9.3	IC_50_ (binding)	pan-RAR (α/β/γ)	active at RARα (≈5.1 nM) and RARβ (≈4.5 nM); classical potent retinoid^23^
LY2955303	antagonist	1.09	*K*_i_ (binding)	highly RARγ-selective	most potent and selective RARγ antagonist in this set^24^
AGN194310	antagonist	2.5	ED_50_	pan-RAR (α/β/γ)	very potent but nonselective across RAR subtypes^25^
AGN205728	antagonist	∼3.0	*K*_i_/ED_50_	RARγ-selective	nanomolar antagonist with good subtype selectivity^22^
MM11253	antagonist	44.0	IC_50_	RARγ	weaker antagonist; included as a reference compound^26^

a EC_50_/AC_50_, half-maximal effective/activating concentration; *K*_i_, inhibition constant; IC_50_, half-maximal inhibitory concentration; ED_50_, half-maximal effective dose; n.d., not determined in the cited sources; n.r., not reported in a directly comparable assay.

The reported activity or binding values originate from multiple assay formats and experimental systems. They are therefore used solely to support ligand-set curation and functional annotation rather than for direct quantitative comparison.

The VDR-ligand subset included the endogenous agonist 1,25D3, synthetic agonist-like or partial agonist-like analogues, and structurally distinct antagonists or tissue-selective modulators. These compounds were selected to represent different modes of *h*VDR modulation, including full agonism, attenuated or partial agonist-like behavior, and antagonism associated with altered H12/AF-2 organization. Their chemical structures are presented in Scheme 2, and available potency, binding, and functional information are summarized in Table 2.

**Scheme 2:**
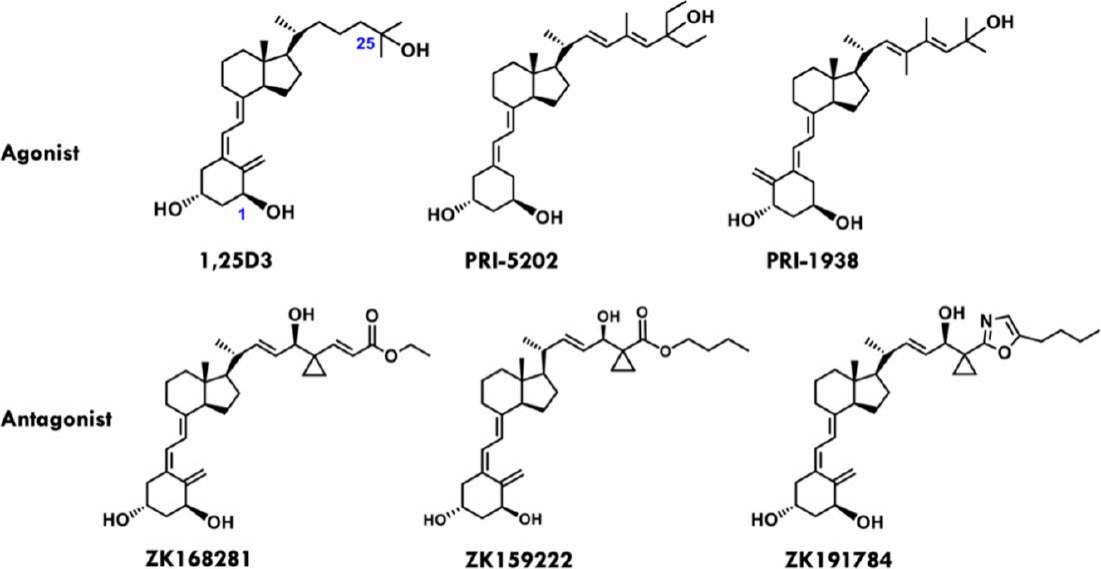
Structures of *h*VDR Ligands Investigated in This Study.

**Table 2:** Reported In Vitro Activity, Binding, or Functional Data for the VDR Ligands Included in This Study.^a^

compound	functional annotation	reported in vitro value (nM)	metric type	selectivity/target profile	comments
1,25D3	agonist (endogenous)	∼0.05–0.5	*K*_d_ (binding)	VDR	high-affinity natural VDR agonist with robust genomic activity^27,28^
PRI-5202	synthetic agonist-like analogue	n.r.	n.r.	VDR	1,25D3 analogue with strong differentiation activity and reduced calcemic effects; directly comparable binding constants were not available^29^
PRI-1938	synthetic partial agonist-like analogue	n.r.	n.r.	VDR	no standardized *K*_i_/EC_50_ value was identified in directly comparable assay conditions
ZK168281	antagonist	∼0.1	*K*_d_ (binding)	VDR	very high-affinity VDR antagonist; inhibits coactivator recruitment^30^
ZK159222	antagonist/partial agonist	∼0.6–1.0	*K*_d_/*K*_i_ (assay-dependent)	VDR	high-affinity VDR ligand; residual partial agonism has been observed in functional assays^30^
ZK191784	tissue-selective modulator	n.r.	n.r.	VDR	intestine-selective antagonist of calcitriol with mixed agonist/antagonist activity in other tissues^31^

a EC_50_, half-maximal effective concentration; *K*_i_/*K*_d_, inhibition/dissociation constant; n.r., not reported in directly comparable assay.

Like the selected RARγ ligands, the reported values for the VDR ligands originate from different experimental systems and are therefore used for functional annotation rather than for direct quantitative ranking.

The central objective of this work is to determine how experimentally annotated ligand classes are reflected in the coupled structural, thermodynamic, and dynamical organization of *h*RARγ and *h*VDR LBDs. Rather than treating binding affinity, MM-PBSA binding free energy, root-mean-square deviation (RMSD), root mean-square fluctuation (RMSF), or individual structural or energetic metrics as standalone predictors of efficacy, we evaluate whether agonist, antagonist, and intermediate ligand states can be distinguished by integrated patterns of local stabilization, ligand–receptor interaction networks, and H12-centered AF-2 organization. The study applies a unified comparative framework to carefully curated *h*RARγ and *h*VDR-ligand sets, enabling ligand efficacy to be interpreted through combined thermodynamic and conformational signatures rather than affinity alone. Within this framework, the MM-PBSA-derived effective enthalpy-like term is interpreted as an apparent energetic descriptor of ligand-dependent stabilization, whereas the residual apparent entropic term is treated strictly as a qualitative descriptor rather than as an independent calculated estimate of configurational entropy. Accordingly, this work is presented as a retrospective mechanistic benchmark for interpreting functional ligand classes in NR systems, rather than as an externally validated predictive classifier. Together, these analyses provide an internally consistent basis for understanding how ligand-dependent structural, energetic, and dynamical features contribute to functional selectivity in *h*RARγ and *h*VDR.

## 2 Materials and Methods

### 2.1 MicroED Data Collection and Structure Determination

Samples were deposited onto freshly glow-discharged, 200-mesh Cu lacey-carbon grids under ambient conditions. The grids were clipped and transferred into a Thermo Fisher Scientific Glacios 200 kV field-emission gun cryo-transmission electron microscope (cryo-TEM) operated by the Cryomicroscopy and Electron Diffraction Core Facility at the Centre of New Technologies, University of Warsaw, Warsaw, Poland. During data acquisition, samples were maintained at 80 K under high vacuum (8 × 10^–6^ Pa). Suitable single-crystalline domains were identified, and electron diffraction data sets were collected under parallel illumination using EPU-ED software with a Ceta-D detector operating in 2 × 2 binning mode (pixel size: 28 μm). The effective camera length was adjusted according to the diffraction limit of the crystal, and an electron wavelength of 0.025 Å was applied. Continuous-rotation MicroED data were acquired over a tilt range from −60° to 60° with a rotation increment of 0.5° per frame (240 frames total) and an exposure time of 0.5 s per frame. The condenser aperture was set to 50 μm, with a spot size of 11 and a gun lens setting of 8, yielding an electron flux density of 0.036 eÅ^–2^ s^–1^. Diffraction frames were processed in CrysAlisPro,^32^ and unit-cell parameters were determined by least-squares refinement against optimized reflection positions. Data reduction and scaling were performed, and the highest-quality data sets were merged to improve completeness. Structure solution was carried out in Olex2 ^33^ using SHELXT^34^ via intrinsic phasing, followed by full-matrix least-squares refinement in SHELXL.^35^ Refinements employed UCLA-2022 (4G)^36^ electron scattering factors in the kinematic approximation, with extinction corrections applied to partially account for dynamical scattering effects.

### 2.2 Ligand Docking to Nuclear Receptors

Molecular docking of agonists and antagonists to the LBDs of *h*RARγ and *h*VDR (Schemes 1 and 2) was performed using GOLD Hermes 2024.3.0 (Genetic Optimization for Ligand Docking).^37^ GOLD employs a genetic algorithm to explore ligand conformational and orientational space within the binding pocket, enabling flexible sampling of ligand rotatable bonds and optional side-chain flexibility of selected receptor residues. The algorithm represents candidate ligand poses as a population of solutions encoded by torsional, translational, and rotational parameters, which are iteratively optimized according to the scoring function.

Receptor structures used for docking were selected from the RCSB Protein Data Bank (https://www.rcsb.org/, accessed on July 15, 2025) based on the following criteria: high crystallographic resolution, human origin, completeness of the modeled LBD region, the absence of reported mutations, and the presence of a cocrystallized ligand defining the canonical binding pocket. For *h*RARγ, the agonist-bound LBD complex with CD564 was selected as the reference structure (PDB ID: 1FCY; 1.30 Å). For *h*VDR, the agonist-bound LBD complex with the natural ligand 1,25D3 was used (PDB ID: 1DB1; 1.80 Å). These structures were selected as high-resolution, experimentally characterized human NR LBD templates suitable for defining the docking site and performing receptor-specific redocking validation. Detailed information on resolution, mutation status, and modeled-residue completeness is provided in Table S1. As no antagonist-bound *h*RARγ LBD structure corresponding to the analyzed antagonist set was available, the agonist-bound *h*RARγ structure was used as a common template for both agonists and antagonists. This approach enabled all ligands within each receptor subset to be evaluated under consistent structural conditions, while acknowledging that docking to a single receptor conformation does not fully capture ligand-induced receptor plasticity. Four GOLD scoring functions (ChemScore, ChemPLP, GoldScore, and ASP) were evaluated in receptor-specific redocking tests for their ability to reproduce experimental ligand poses. The full redocking results are summarized in Table S2 and Figure S1. Based on the combined score/RMSD performance, ChemPLP was selected as the primary scoring function for subsequent docking calculations because it showed the most consistent overall performance across the reference systems. To validate the docking workflow, redocking of the cocrystallized reference ligands was performed, and the resulting poses were compared with the corresponding crystallographic conformations. For the *h*RARγ agonist reference complex, ChemPLP reproduced the crystallographic pose with an RMSD of 0.30 Å, whereas for the *h*VDR agonist reference complex, the corresponding RMSD was 1.38 Å. Thus, pose reproduction was more accurate for *h*RARγ than for *h*VDR, although the *h*VDR result remained within an acceptable range for docking-based pose recovery. Importantly, docking scores were used to rank poses within each receptor–ligand system and were not interpreted as directly comparable between *h*RARγ and *h*VDR. On this basis, ChemPLP was used for subsequent docking calculations in both receptor systems.

### 2.3 Partial Charge Assignment and Ligand Force-Field Parameterization

All agonists and antagonists of the analyzed NRs were prepared before docking and MD simulations. For ligands with available receptor-bound crystal structures, including LY2955303, ZK168281, ZK159222, and 1,25D3, the ligand geometries were extracted from the corresponding high-resolution structures deposited in the RCSB Protein Data Bank. For AGN205728 and AGN194310, experimentally determined MicroED structures obtained in the present study were used as structural references, as described in Sections 3.1 and 3.2. For the remaining ligands, including AGN204647, AGN191183, MM11253, AGN205327, palovarotene, CD1530, ZK191784, PRI-5202, and PRI-1938, initial three-dimensional structures were obtained from PubChem (https://pubchem.ncbi.nlm.nih.gov/) or generated in silico based on structurally related analogues. The ligand geometries were optimized using density functional theory (DFT) implemented in Gaussian 16.^38^ Geometry optimization and electrostatic potential (ESP) calculations were performed at the B3LYP/6-311G++(d,p) level of theory. Atomic partial charges were derived by fitting point charges to the quantum-mechanical electrostatic potential using the ChelpG method.^39^ For each ligand, the quality of the ESP-derived partial charges was assessed using the RMS and relative RMS error of the electrostatic potential fit, along with verification of the total molecular charge and the sum of the fitted ESP charges. These charge-fit quality metrics are reported in Table S3. The resulting ESP-derived partial charges were assigned to all of the ligand atoms and used for subsequent molecular modeling and MD simulations. Ligand force-field parameters were generated within the CHARMm framework^40^ using Discovery Studio v22.1 ^41^ to ensure compatibility with the protein force field employed in the simulations. Missing bonded and nonbonded parameters were assigned by analogy to existing CHARMm atom types and manually inspected for chemical consistency. This workflow provided a consistent description of ligand electrostatics and intramolecular interactions for docking, MD simulations, and MM-PBSA calculations.

### 2.4 Molecular Dynamics Simulations of *h*RARγ- and *h*VDR-Ligand Complexes

MD simulations of all docked receptor–ligand complexes were carried out using the CHARMm force field as implemented in the Discovery Studio software suite. Each complex was solvated in a cubic box of TIP3P water molecules^42^ extending at least 10 Å from any solute atom. Counterions (Na^+^ and Cl^–^) were added to neutralize the systems and to obtain an ionic strength of approximately 0.15 M, corresponding to physiological conditions, using the Solvation Module implemented in Discovery Studio. Periodic boundary conditions were applied, and long-range electrostatic interactions were treated using the particle mesh Ewald (PME) method.^43^ Prior to MD simulations, all systems were subjected to energy minimization consisting of 10,000 steps of steepest descent followed by 20,000 steps of conjugate gradient minimization, until the root-mean-square (RMS) gradient fell below 0.01 kcal·mol^–1^·Å^–1^. During minimization and equilibration, harmonic positional restraints of 2 kcal·mol^–1^·Å^–2^ were applied to the protein backbone atoms, with the exception of helices H11 and H12, which were left unrestrained to allow conformational relaxation of the AF-2 region. An additional unrestrained conjugate gradient minimization of 1000 steps was subsequently performed. The systems were then gradually heated from 50 to 300 K over 100 ps, followed by equilibration at 300 K for 200 ps. The resulting equilibrated structures served as starting points for production simulations. For each receptor–ligand complex, three independent MD production runs were performed, each initiated with a different random velocity seed. Each replicate had a total duration of 100 ns and was conducted in two consecutive stages: an initial 50 ns simulation in the canonical (NVT) ensemble at 300 K with weak harmonic restraints applied to the solute, followed by a 50 ns unrestrained simulation in the isothermal–isobaric (NPT) ensemble at 300 K and 1 atm. Temperature control was achieved using a Langevin thermostat,^44^ with a temperature coupling decay time of 5 ps and a friction coefficient (γ) of 5, as defined in the Discovery Studio CHARMm protocol. During the NPT phase, pressure was regulated using the CHARMm constant pressure/temperature (CPT) algorithm with the CPT mass set to 1000 and p_γ_ equal to 20. All simulations employed the SHAKE algorithm to constrain bonds involving hydrogen atoms, allowing for a 2 fs integration time step.^45^ The cutoff distance of 12 Å was used for nonbonded interactions, and trajectory snapshots were saved every 1 ps. Structural stability of the simulated complexes was assessed by calculating root-mean-square deviation (RMSD) values along the MD trajectories after alignment to the initial reference structure using the receptor backbone Cα atoms. RMSD profiles were analyzed to evaluate equilibration behavior and overall complex stability. Frames extracted at 50 ps intervals were used for root-mean-square fluctuation (RMSF) and receptor–ligand contact analyses. Receptor–ligand interactions were quantified based on donor–acceptor distances (Å).

### 2.5 Binding Free-Energy Estimation Using Molecular Mechanics Poisson–Boltzmann Surface Area (MM-PBSA)

Binding free energies were estimated using the MM-PBSA approach^46^ as implemented in Discovery Studio v22 to obtain a comparative energetic description of agonist- and antagonist-bound complexes of *h*RARγ and *h*VDR. Calculations were performed on the equilibrated portion of the production trajectories using the last 200 snapshots extracted from each 100 ns production replicate. Binding free energies were computed according to Δ*G*_bind_ = *G*_complex_ – *G*_receptor_ – *G*_ligand_, where *G*_complex_, *G*_receptor_, and *G*_ligand_ represent the free energies of the receptor–ligand complex, isolated receptor, and isolated ligand, respectively. Each free-energy term was decomposed into molecular mechanics contributions, including electrostatic and van der Waals interactions, along with polar solvation energies computed using the Poisson–Boltzmann continuum model and nonpolar solvation energy estimated from the solvent-accessible surface area. The PBSA calculations employed a solute dielectric constant of ε_solute_ = 4 and an implicit solvent dielectric constant of ε_solvent_ = 80. A solute dielectric constant of 4 was used to represent a partially polarizable protein–ligand interior and to reduce the overemphasis of gas-phase electrostatic interactions inherent to the fixed-charge CHARMm force-field framework, whereas a solvent dielectric constant of 80 represents a bulk aqueous solvent. The nonpolar solvation contribution was estimated using a solvent-accessible surface area term with a surface coefficient of 5.42 × 10^–3^ kcal mol^–1^ Å^–2^ and a surface constant of 0.92 kcal mol^–1^. Salt concentration was set to zero, atomic radii were assigned from van der Waals radii, and molecular surfaces were used in the PBSA calculations. A nonbonded list radius of 14 Å and spherical cutoff electrostatics were applied. All parameters were kept identical for all receptor–ligand complexes to ensure an internally consistent comparison across the ligand panel. On this basis, the effective energetic component reported in this work was defined as Δ*H*_eff_ = Δ*E*_vdW_ + Δ*E*_elec_ + Δ*G*_polar_ + Δ*G*_nonpolar_. This quantity should be understood as an operational MM-PBSA energetic descriptor of ligand-dependent stabilization, rather than as a directly measurable calorimetric enthalpy. In particular, because continuum solvation free-energy terms contain both enthalpic and entropic contributions, their inclusion in Δ*H*_eff_ renders this term an effective enthalpy-like descriptor rather than a strict thermodynamic Δ*H*. No explicit configurational entropy contribution was included in the MM-PBSA analysis. An apparent residual entropy-related term was defined operationally as the difference between the binding free energy and the effective enthalpic component: −*T*Δ*S*_app_ = Δ*G*_bind_ – Δ*H*_eff_. This term was treated solely as a qualitative descriptor within the MM-PBSA framework. Accordingly, the MM-PBSA analysis was used to identify comparative energetic trends between ligand classes within an internally consistent computational framework rather than to determine absolute binding free energies or to establish an externally validated predictive classification model.

### 2.6 Internal Descriptor-Separability Assessment

To compare the ability of Δ*G*_bind_ and Δ*H*_eff_ to distinguish agonist- and antagonist-bound complexes within the present ligand set, an internal retrospective descriptor-separability assessment was performed. This analysis follows the general principle of threshold-based separation of continuous descriptors commonly used to evaluate whether a quantitative variable can discriminate between two predefined classes.^47,48^ For each receptor–ligand complex, a single descriptor value was used, calculated as the mean across three independent MD replicas. Functional labels (agonist or antagonist) were assigned based on experimentally reported ligand classification. Descriptor-based separation was evaluated in one dimension for each descriptor independently. Because more favorable binding or enthalpic stabilization corresponds to more negative values, boundary descriptor values were defined as the least favorable agonist value and the most favorable antagonist value within the data set. The decision threshold was placed at the midpoint between these two boundary values:

$$t=\frac{x_{\mathrm{ag},\max}+x_{\mathrm{ant},\min}}{2}$$

where *x*_ag,max_ represents the least favorable agonist value and *x*_ant,min_ denotes the most favorable antagonist value. Ligands with descriptor values equal to or more favorable (i.e., more negative) than the threshold were considered concordant with agonist-like behavior, whereas ligands with less favorable values were considered concordant with antagonist-like behavior. The apparent internal assignment was then calculated as the fraction of ligands whose descriptor values were concordant with their experimentally reported functional classification. To quantify the extent of separability between the two functional groups, the minimum interclass descriptor gap was defined as

$$g=x_{\mathrm{ant},\min}-x_{\mathrm{ag},\max}$$

Positive values of *g* indicate a finite separation between the agonist- and antagonist-associated descriptor ranges, whereas values close to zero or negative values indicate partial or complete overlap. As an internal robustness check, a leave-one-ligand-out threshold analysis was additionally performed. In this procedure, each ligand was removed in turn, the decision threshold was recalculated using the remaining ligands, and the omitted ligand was evaluated against the recalculated threshold. This procedure was used solely to assess the stability of the observed internal separation and was not intended as an external validation. Accordingly, this analysis was used exclusively to compare the relative internal separability of Δ*G*_bind_ and Δ*H*_eff_ within the present ligand panel and was not designed to establish a prospective predictive classification model. This limitation is consistent with general recommendations for descriptor-based approaches, for which independent external validation is required prior to predictive application.^49^ In addition, group-level differences between agonist- and antagonist-bound complexes were evaluated using Welch’s two-tailed *t*-test, using a single mean descriptor value per receptor–ligand complex as the statistical unit.

## 3 Results and Discussion

### 3.1 Structural Organization and Packing Characteristics of AGN194310

The MicroED structure of AGN194310 illustrates how sterically demanding molecular architectures can still adopt highly ordered arrangements at the nanoscale. Despite the large molecular size, the compound crystallizes in the monoclinic *P*2_1_/*c* space group with two crystallographically independent molecules in the asymmetric unit, reflecting conformational adaptability rather than static rigidity. The absence of atomic disorder within the molecules of the studied compound suggests that intermolecular stabilization is dominated by cumulative weak interactions rather than a single directional motif. However, solvent-accessible voids revealed during refinement, corresponding to approximately 22.7 e within a volume of 172.5 Å^3^ suggest the presence of disordered solvent molecules within the crystal structure (Table S4). Such cavities indicate a relatively open packing arrangement, consistent with an elongated and nonplanar molecular framework. From a crystal engineering perspective, this packing inefficiency may originate from steric shielding by peripheral substituents that hinder dense π–π stacking. The resulting porosity influences diffusion properties or guest accommodation, suggesting that AGN194310 represents a structural motif in which weak dispersive contacts outweigh classical hydrogen bonding in defining lattice stability (Figure 2).

**Figure 2:**
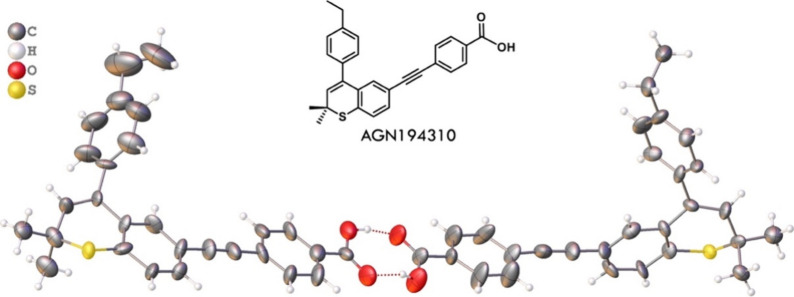
Asymmetric unit of AGN194310 viewed along the *a* axis represented in a balls and sticks model. Ellipsoids of nonhydrogen atoms are drawn at the 50% probability level.

Analysis of molecular packing (Figure S2) reveals that crystal cohesion is governed primarily by van der Waals interactions and aromatic contacts rather than strong directional hydrogen bonds. This contrasts with the behavior of AGN205728 and highlights how subtle functional group variation drastically alters supramolecular assembly.

### 3.2 Hydrogen-Bond-Driven Polymorphism of AGN205728

In contrast to AGN194310, AGN205728 exhibits pronounced polymorphism arising from competition between alternative hydrogen-bonding patterns. Two polymorphic forms, denoted α and β, were resolved by MicroED. Both crystallized in the triclinic *P-*1 space group, each with two molecules in asymmetric unit, but adopted distinct supramolecular organizations (Figure 3). The α-form is characterized by homodimeric assemblies in which NOH···NOH and COOH···COOH interactions generate symmetric hydrogen-bonding motifs. These interactions propagate into extended B–A–B chains (Figure S3), producing an open packing arrangement that accommodates conformational variability between the two independent molecules.

**Figure 3:**
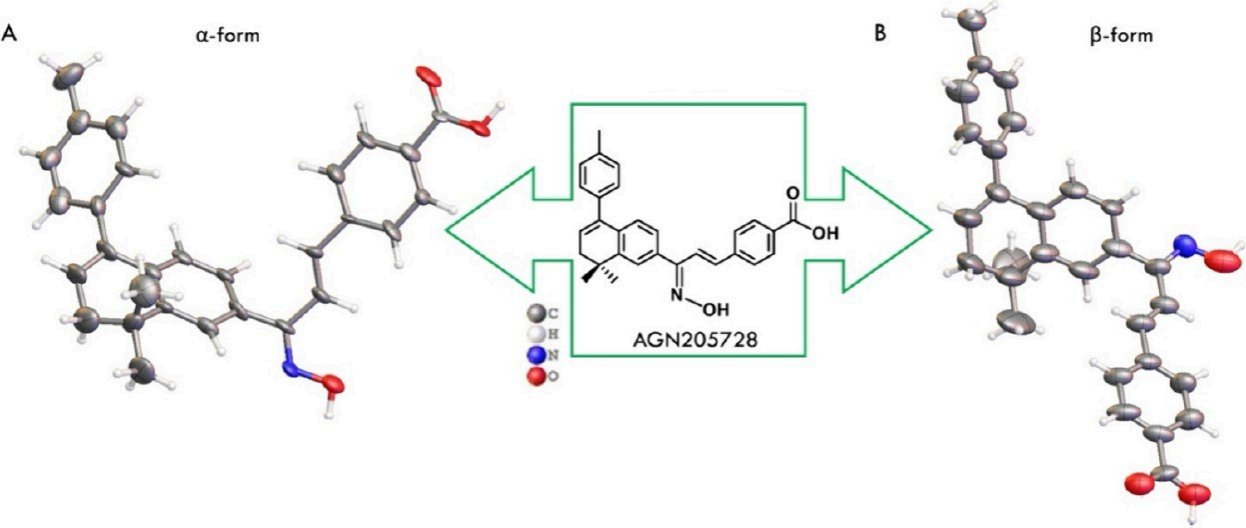
Half of the asymmetric units of AGN205728 polymorphs: (A) α-form and (B) β-form. Ellipsoids of nonhydrogen atoms are drawn at the 50% probability level.

The ability to locate all hydrogen atoms directly from difference Fourier maps indicates a well-defined hydrogen-bonding geometry and highlights the quality of the electron diffraction data. By contrast, the β-form exhibits heterodimeric interactions in which NOH groups donate to COOH acceptors and vice versa. This inversion of donor–acceptor roles reorganizes the packing into a less compact lattice, reflected in the decreased calculated density (1.19 g cm^–3^) relative to the α-form (1.24 g cm^–3^) (Table S5). Such density differences, although modest, suggest that the β-form may represent a thermodynamically less efficient crystal packing arrangement. Molecules A and B (Figure S3) retain similar core geometries but differ in peripheral torsion angles, underscoring conformational flexibility as a key driver of polymorphism. Rather than a drastic molecular rearrangement, polymorphic diversity emerges from subtle reorientation of hydrogen-bond donors and acceptors, demonstrating how minimal conformational changes can trigger large-scale reorganization of the crystal lattice. Direct comparison of B–A–B chains reveals that the two polymorphs are related by a supramolecular "switch" in hydrogen-bond connectivity. In the α-form, homodimeric motifs promote extended linear arrangements, whereas heterodimeric interactions in the β-form induce a more folded topology. This transformation highlights the dual role of NOH and COOH functionalities as adaptable supramolecular synthons capable of stabilizing multiple packing motifs (Figure S4). From a crystal engineering standpoint, such behavior exemplifies competitive hydrogen bonding, where multiple energetically comparable arrangements coexist. The presence of two polymorphs within the same structural family suggests a shallow energy landscape, where small environmental variations during crystallization may dictate the final packing mode.

### 3.3 Structural Analysis of Differential Interaction Patterns of *h*RARγ Agonists and Antagonists

Agonist and antagonist ligands were docked into the ligand-binding pocket of *h*RARγ (Figure 4 and Table S6). Although both agonists (AGN205327, AGN191183, AGN204647, CD1530, and palovarotene) and antagonists (AGN205728, AGN194310, MM11253, and LY2955303) occupy the same ligand-binding cavity, they establish distinct interaction networks within the *h*RARγ LBD, which are associated with different functional outcomes. The interaction features identified in the present docking and MD analyses were interpreted within the established structural framework of *h*RAR and NR activation. Agonist binding is known to promote active-like positioning of H12 and formation of the AF-2 coactivator-binding surface, whereas antagonist binding often involves bulky or posteriorly extended ligand groups that interfere with the H11/H12 region and perturb H12 organization.^8,50,51^ Together, Table 3, Table S7, and Figure S5A integrate key interaction patterns that distinguish *h*RARγ agonists from antagonists and support a mechanistic interpretation consistent with the literature, linking posterior ligand engagement to altered H12-adjacent packing and AF-2-incompatible or perturbed receptor organization.^9^

**Figure 4:**
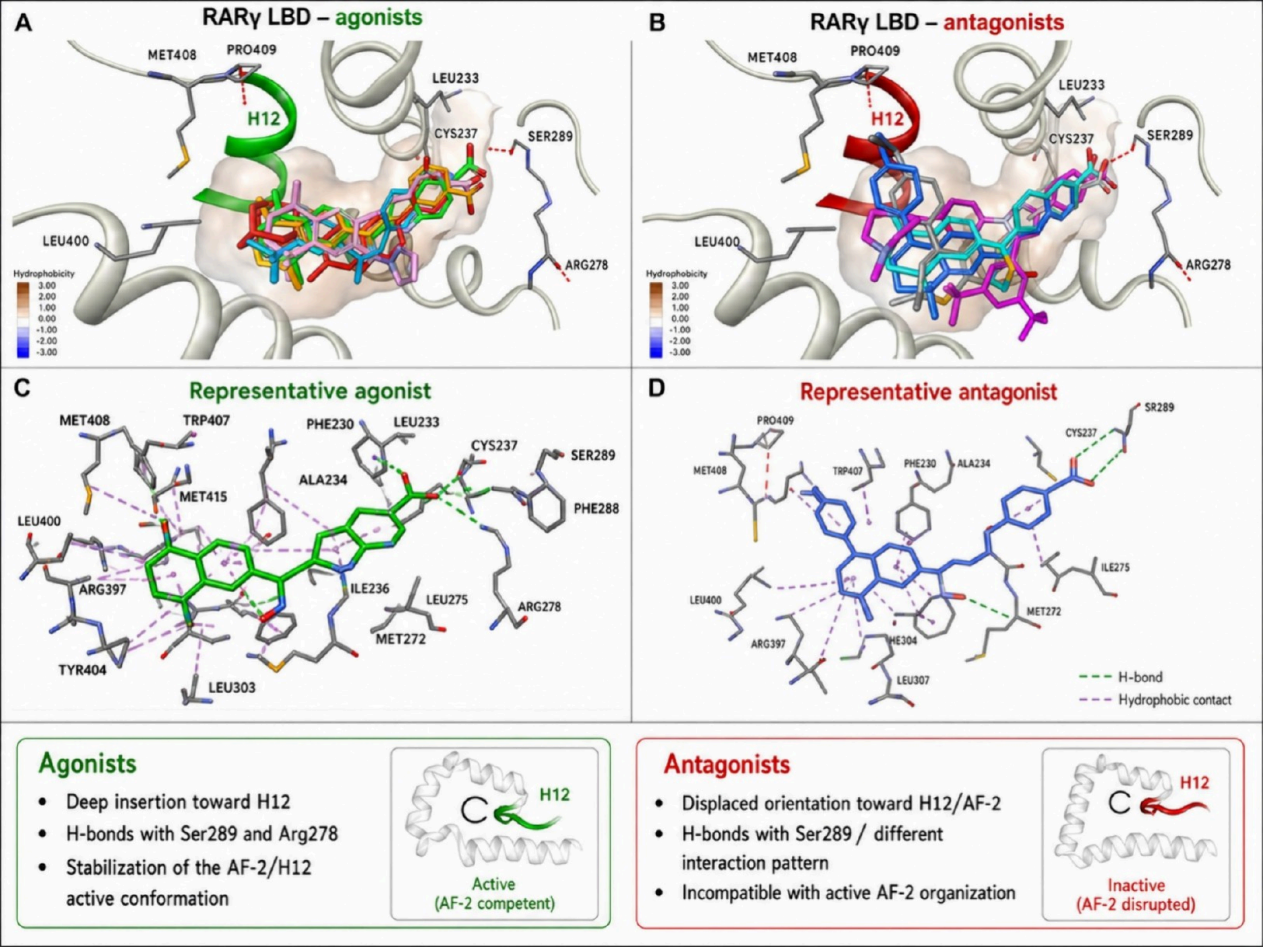
Comparative binding modes of *h*RARγ agonists and antagonists. (A) Superposition of agonists within the *h*RARγ ligand-binding pocket. (B) Superposition of antagonists in the same binding pocket and orientation as in panel (A). (C) Close-up of a representative agonist, AGN205327, highlighting key ligand–receptor interactions that stabilize the activation-compatible pocket conformation. (D) Close-up of a representative antagonist, AGN205728, illustrating distinct interaction patterns associated with antagonist-like modulation. The receptor is shown as a gray cartoon representation; ligands are depicted as sticks, with selected pocket residues shown as labeled sticks. Hydrogen bonds and hydrophobic contacts are indicated by dashed lines. Color coding of ligands: AGN205327 (green), AGN191183 (blue), AGN204647 (orange), CD1530 (red), palovarotene (pink), AGN205728 (dark blue), AGN194310 (gray), MM11253 (turquoise), and LY2955303 (magenta).

**Table 3:** Docking- and MD-Derived Interaction Features That Distinguish *h*RARγ Agonists and Antagonists and Their Proposed Mechanistic Interpretation.

feature	agonists	antagonists	mechanistic relevance
Arg278	direct hydrogen bonds frequently observed	absent or markedly reduced	polar anchoring interaction supporting anterior ligand orientation^8,9^
Ser289/Phe288	hydrogen bonds	hydrogen bonds, often water-mediated	secondary polar anchoring; maintained in both classes^8,9^
Cys237	π-sulfur/alkyl contacts	π-sulfur/alkyl contacts	supportive interaction observed in both classes; not functionally discriminative within the present data set
ligand position	anterior/central pocket	posteriorly shifted	determines accessibility of the H11/H12 region^50,51^
core hydrophobic cage	conserved (Leu268, Leu271, Ile275, Phe230, Met272, Ala234, Ala397, and Phe304)	conserved	provides baseline hydrophobic stabilization for all ligands^8,9^
H12-adjacent contacts	Ile412, Met415, and Leu416 with stabilizing geometry	Ile412 contacted with altered geometry	contacts near H12 may contribute to active-like positioning or altered H12 dynamics, depending on ligand geometry^8,50,51^
posterior pocket engagement	minimal	pronounced	posterior occupation by extended antagonist moieties may promote steric interference near H11/H12 and reduce compatibility with the canonical active AF-2 arrangement^50,51^
Phe304	moderate π–π/π-alkyl	strong π–π stacking	aromatic contact stabilizing posterior binding poses; in the present data set, stronger Phe304 engagement is associated with antagonist-like posterior occupation
Trp227	weak or nonspecific	aromatic π–π stacking in MM11253 only	variant-specific posterior pocket stabilization; interpreted as ligand-specific rather than a general antagonist marker
Pro409	absent	auxiliary hydrophobic contacts (AGN205728, AGN194310)	indicative of deep posterior penetration near the H11/H12 region
ligand geometry	compact	elongated/bulky	controls occupation of the H11/H12-adjacent region; bulky extensions may promote antagonist-like H12 repositioning^50,51^
H12-associated effect	supports active-like H12 positioning	destabilized/displaced	contributes to AF-2 surface organization^8,50,51^
AF-2 surface	properly formed	disrupted	governs coregulator recruitment^8,50,51^

MD simulations indicate that the molecular geometry of the α-form of AGN205728 is better accommodated within the *h*RARγ ligand-binding pocket than that of the β-form. Accordingly, the α-form was selected for subsequent docking, MD simulations, and binding-energy analyses. This selection reflects the closer agreement between the α-form geometry and the receptor-bound conformation observed for AGN205728, particularly with respect to the relative orientation of the NOH and COOH functionalities and peripheral substituents, which are positioned favorably for stable accommodation within the ligand-binding site and for the formation of stabilizing receptor–ligand interactions. In contrast, the β-form adopts an alternative molecular arrangement that is less compatible with the steric and interaction constraints of the *h*RARγ binding pocket. The resulting orientation of key functional groups would impose unfavorable geometric constraints for stable binding; therefore, the β-form was not considered in subsequent receptor-bound simulations. Because the β-form corresponds to a solid-state polymorphic arrangement, it should not be interpreted as a polymorphic state expected to appear directly during MD simulations of an isolated protein–ligand complex (Figure S6). Nevertheless, possible β-like ligand conformations were considered during trajectory analysis, and no persistent β-like conformation of AGN205728 was observed in the analyzed trajectories.

This further supports the use of the α-form as the receptor-compatible starting structure. In all agonist complexes (Figure 4A and Figure S7), ligands adopt a compact binding mode anchored by direct polar hydrogen-bonding interactions with Arg278 (H5) and Ser289 (H6), typically complemented by hydrogen bonds to Phe288 (H6) and π-sulfur interactions with Cys237 (H3). This Arg278/Ser289-centered anchoring motif fixes ligand orientation in the anterior region of the pocket and constrains translational freedom. Such polar anchoring and compact ligand accommodation are consistent with previously reported structural descriptions of *h*RARγ ligand recognition, in which polar contacts and ligand-induced organization of the ligand-binding domain are associated with active-like conformation of the receptor.^8,51^ The ligand core is further stabilized by a conserved hydrophobic cage formed by Leu268 (H5), Leu271 (H5), Ile275 (H5), Phe230 (H3), Met272 (H5), Ala234 (H3), Ala397 (H10), and Phe304 (H7) through extensive alkyl and π-alkyl interactions, resulting in tight packing and reduced conformational mobility. Critically, agonists consistently establish stabilizing hydrophobic contacts with residues located in or immediately adjacent to H12, most notably Ile412, Met415, and Leu416. For example, AGN205327 and AGN191183 (pan-RAR) interact directly with Ile412 and Met415 via alkyl interactions, while palovarotene additionally engages Leu416. These interactions are consistent with active-like positioning of H12 over the ligand-binding pocket and with stabilization of the canonical agonist-compatible conformation of the LBD. This configuration supports formation of the AF-2 surface, which is required for efficient coactivator recruitment and transcriptional activation.^8,50,51^ In contrast, none of the antagonists display direct interactions with Arg278 (Figure S7).

Polar anchoring in antagonist complexes is maintained primarily by Ser289, Phe288 and Cys237, frequently involving water-mediated hydrogen bonds. Loss of Arg278 anchoring releases positional constraints on the ligand and permits posterior displacement deeper into the binding pocket. This relocation is accompanied by engagement of residues contacted only weakly or nonspecifically by agonists, together with a posteriorly shifted interaction geometry involving Phe304, Ala397, Leu400 (H11), and Ile412 (H12). All antagonists display elongated or bulky substituents that expand toward the H11/H12 region. AGN205728 and AGN194310 show pronounced π–π stacking interactions with Phe304, accompanied by extensive alkyl contacts with Ala397 and Leu400, whereas MM11253 additionally exhibits a well-defined aromatic π–π interaction with Trp227 (H3) (Figure 4B and Figure S8). In the AGN205728 and AGN194310 (pan-RAR) antagonist complexes, auxiliary hydrophobic contacts with Pro409 (located at the H11–H12 junction) are also observed, consistent with posterior ligand displacement rather than a specific anchoring interaction. These posterior contacts preferentially occupy the H11/H12 region, which may interfere with active-like positioning of H12. Rather than stabilizing the active conformation, antagonist binding is predicted to alter H12 dynamics and reduce compatibility with the formation of the active AF-2 surface, shifting the receptor toward a less coactivator-compatible and potentially corepressor-compatible state.^50,51^ Thus, antagonism in RARγ does not arise from occupation of a distinct binding site but from ligand-induced remodeling of the interaction network surrounding helices H11 and H12. The decisive mechanistic step appears to be the loss of Arg278 anchoring, which permits posterior ligand migration and subsequent alteration of H12-associated interactions. To illustrate the structural consequences of this mechanism, the binding poses of a representative agonist (AGN205327) and antagonist (AGN205728) were superposed within the *h*RARγ LBD (Figure 5). As shown in Figure 5, the agonist adopts an anterior binding orientation that supports interactions stabilizing the H12 region, whereas the antagonist is displaced toward the posterior part of the pocket, occupying the H11/H12 region and potentially interfering with the positioning of helix H12. Therefore, *h*RARγ agonists act through a compact binding mode anchored by Arg278, which enforces a ligand orientation conducive to dense hydrophobic packing and direct stabilization of Ile412, Met415, and Leu416. This interaction network supports active-like H12 positioning, enabling AF-2 formation and coactivator recruitment. In contrast, *h*RARγ antagonists lack Arg278 anchoring and adopt a posteriorly shifted binding pose characterized by engagement of Trp227 and Pro409 in selected cases, together with an altered interaction geometry involving Phe304, Ala397, residues on helix H11 (Leu400), and helix H12 (Leu412). These interactions favor occupation of the H11/H12 region that is less compatible with stable H12 positioning, thereby perturbing AF-2 assembly and favoring an antagonist-like receptor state.

**Figure 5:**
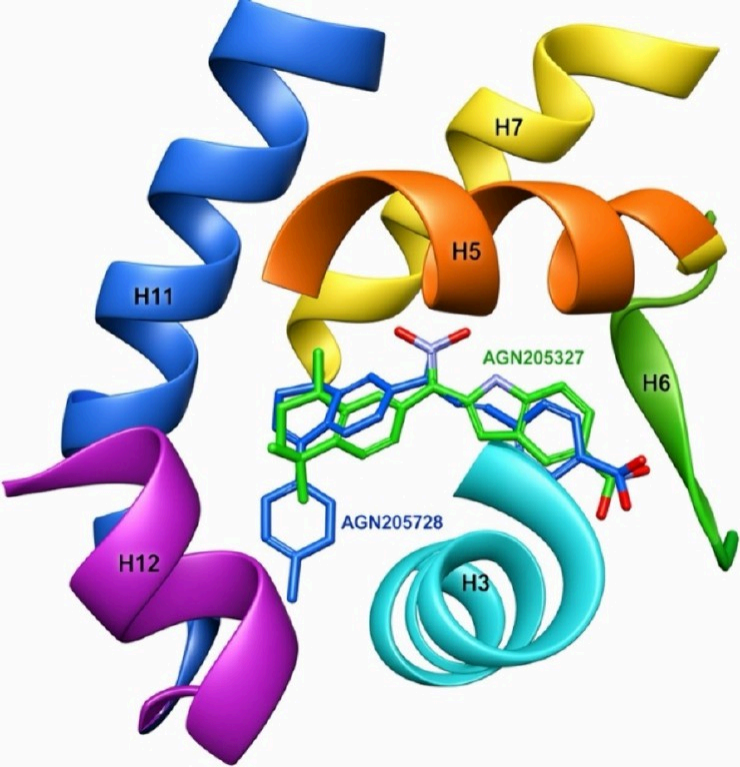
Comparison of representative agonist and antagonist binding modes in the *h*RARγ ligand-binding domain. Representative conformations of AGN205327 and AGN205728 are shown within the binding pocket. Helices H3, H5, H6, H7, H11, and H12 are indicated to provide structural reference and spatial orientation.

Taken together, these findings demonstrate that functional selectivity in *h*RARγ is governed primarily by ligand-controlled occupation of the H11/H12 region rather than by differences in the binding site location.

### 3.4 Structural Analysis of Differential Interaction of *h*VDR Agonists and Antagonists

The present study provides a comparative structural analysis of *h*VDR agonists and antagonists based on molecular docking and MD simulations. By systematically examining ligand–receptor interactions, we identified key molecular determinants discriminating agonistic and antagonistic behavior and rationalized the functional profiles of the ligands. Agonists (1,25D3, PRI-5202, and PRI-1938) and antagonists (ZK168281, ZK159222, and ZK191784) were docked into the ligand-binding pocket of the *h*VDR, showing their predicted binding modes (Figure 6 and Table S8).

**Figure 6:**
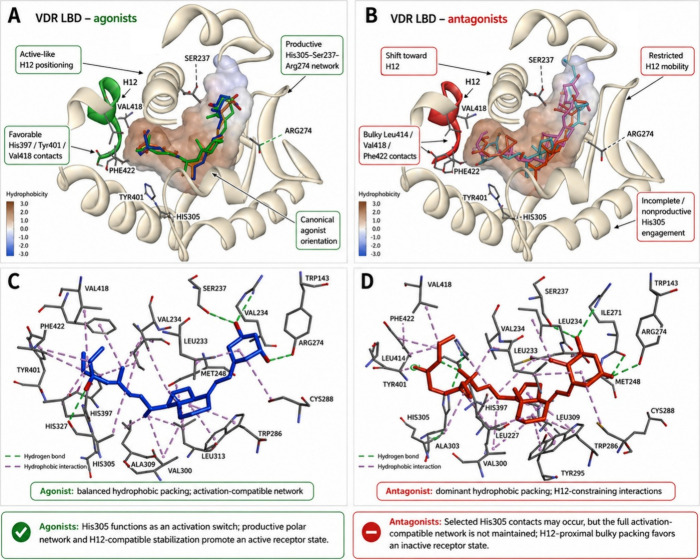
Comparison of *h*VDR agonist/agonist-like and antagonist binding modes and their proposed effects on H12-associated receptor organization. (A) Overlay of *h*VDR LBD agonist/agonist-like complexes showing canonical ligand orientation and active-like H12 positioning, with H12 highlighted in green. (B) Overlay of *h*VDR LBD antagonist complexes showing ligand displacement toward the H12 region and antagonist-associated H12 restriction, with H12 highlighted in red. (C) Detailed interaction pattern for the representative agonist-like ligand PRI-5202, showing Ser237/Arg274-mediated polar anchoring and H12-compatible hydrophobic packing. (D) Detailed interaction pattern for the representative antagonist ZK168281, showing H12-proximal hydrophobic packing and incomplete or nonproductive His305-associated engagement. Hydrogen bonds are shown as green dashed lines and hydrophobic interactions as purple dashed lines. Superposition of docked ligands: 1,25D3 (green), PRI-5202 (blue), PRI-1938 (orange), ZK168281 (red), ZK159222 (pink), and ZK191784 (turquoise).

The endogenous 1,25D3 agonist adopts the canonical binding mode characterized by a conserved network of hydrogen bonds with Ser237 (H3), Arg274 (H5), and His305 (H7). These residues are widely recognized as key anchoring elements of the *h*VDR LBD. The resulting interactions stabilize the agonist in the proper positioning of H12, a critical requirement for coactivator recruitment and subsequent transcriptional activation (Figure S9). In addition, 1,25D3 forms extensive hydrophobic contacts with residues lining the binding pocket, including Leu227 and Val234 (H3), Trp286 (H6), Val300 (H7), His397, Tyr401 (H11), and Val418 (H12), thereby reinforcing the active receptor conformation. Both PRI-5202 and PRI-1938 engage the LBD of *h*VDR in a manner consistent with the canonical agonist binding mode of 1,25D3. In both complexes, a conserved triad of hydrogen-bonding interactions anchors the ligand hydroxyls: the 1-OH interacts with Ser237 (H3) and Arg274 (H5), the 3-OH with Ser278 (H5) and Tyr143 (loop between H1 and H2), and the 25-OH with His305 (loop between H6 and H7) and His397 (H11). This polar interaction network positions the ligand along the canonical anchoring axis characteristic of activation-competent binding geometries. PRI-5202 maintains a compact interaction topology that closely resembles that of 1,25D3. In addition to the conserved hydrogen bonds, extensive hydrophobic contacts are made with Leu233, Val234, and Ala231 (H3), Ile271 (H5), Cys288 and Trp286 (H6), and Met338 (H8) residues, including π–π stacking interactions involving Trp286. These contacts form a cohesive hydrophobic envelope within the LBD core, showing efficient pocket accommodation without introducing steric perturbations near the H11–H12 region. PRI-1938 preserves the canonical hydrogen-bonding network but exhibits a more expanded interaction. Additional contacts with Met272 (H5) and Cys288 residues are maintained, but the hydrophobic packing appears slightly less compact than that of PRI-5202, reflecting the structural modifications of the 5,6-trans geometry and branched side chain. Despite these differences, the overall interaction pattern remains consistent with agonist-like binding poses. Importantly, interaction analysis alone does not permit direct classification of ligand function. While both analogues adopt binding geometries compatible with an activation-competent orientation of H12, functional agonism in nuclear receptors depends primarily on ligand-induced modulation of conformational dynamics rather than on the presence or number of individual contacts. In contrast, the antagonist ZK168281 displays a markedly different interaction. Most notably, it lacks hydrogen bonding with His305, a residue critically involved in agonist-induced activation. Instead, ZK168281 relies predominantly on hydrophobic and π-alkyl interactions with residues located near H12, including Leu414, Val418, and Phe422 (Figure S9). Superposition of the VDR LBD bound to ZK168281 (antagonist) and PRI-5202 (agonist) (Figure 7) further illustrates the conformational differences induced by antagonists versus agonists.

**Figure 7:**
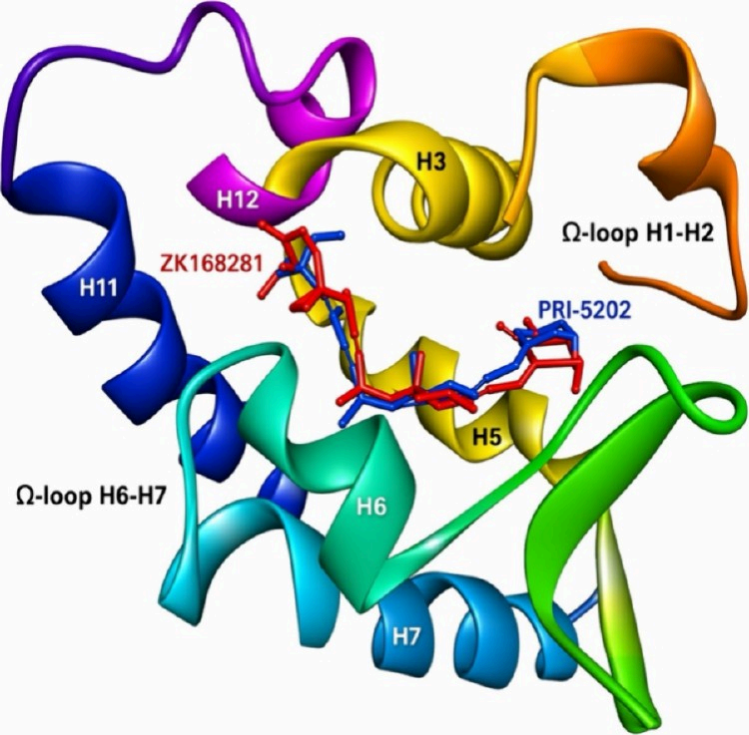
Comparison of representative agonist and antagonist binding modes in the *h*VDR-ligand-binding domain. Representative conformations of PRI-5202 and ZK168281 are shown within the binding pocket. Helices H3, H5, H6, H7, H11, and H12, as well as the Ω-loop H1–H2 and Ω-loop H6–H7, are indicated for structural reference and spatial orientation.

These contacts are consistent with the steric stabilization of an inactive H12 orientation, thereby preventing coactivator binding. ZK159222 presents an intermediate but ultimately antagonistic profile. Although hydrogen bonds with Ser237 and Arg274 are retained, they are insufficient to compensate for the absence of a stabilizing agonist-like network involving His305 and proper alignment of H12. The predominance of hydrophobic interactions and lack of productive contacts within the activation hotspot suggest that functional antagonism or, at most, very weak partial agonism likely translates into antagonistic behavior in cellular assays. ZK191784 further reinforces this trend. This ligand fails to establish the conserved agonist hydrogen-bonding network and instead interacts extensively with hydrophobic residues within the LBD. While hydrogen bonding with His397 is observed, this interaction alone is insufficient to promote the active conformation and is not characteristic of agonist binding. The overall interaction is consistent with the disruption of agonist-induced conformational changes and supports ZK191784 as an *h*VDR antagonist. These results and the reported structural data^52^ indicate that His305 is a key player in coordinating ligand interactions and stabilizing active conformations of the *h*VDR LBD. His305 may act as a key interaction node that contributes to activation-compatible organization of the *h*VDR LBD. Mechanistically relevant interactions involving His305, together with Ser237 and Arg274, may contribute to the ligand-dependent modulation of the H12 region. The interaction patterns that distinguish *h*VDR agonist/agonist-like and antagonist-bound complexes are summarized in Table 4, Table S9, and Figure S5B. These findings highlight the importance of the His305-Ser237-Arg274 interaction network, together with H12-compatible ligand positioning, in regulating *h*VDR activation.

**Table 4:** Mechanistically Relevant Interactions Distinguishing *h*VDR Agonists and Antagonists.

interaction	*h*VDR agonists	*h*VDR antagonists
hydrogen bonding to His305	maintained in the reference agonist 1,25D3 and partially preserved in agonist-like binding modes through compatible Ser237/Arg274 anchoring and H12 positioning	incomplete or geometrically nonproductive; selected His305 contacts may occur, but they do not preserve the full activation-compatible network
His305-Ser237-Arg274 network	geometrically compatible with active-like VDR organization	disrupted, incomplete, or not coupled to productive H12 positioning
role of His305	functions as a molecular activation switch	the activation switch remains inactive
H12 positioning	stabilized in the active conformation, enabling coactivator binding	destabilized or sterically blocked, preventing coactivator recruitment
H12-adjacent residues	favorable contacts with His397, Tyr401, and Val418 supporting activation	bulky hydrophobic interactions with Leu414, Val418, and Phe422 restricting H12 mobility
ligand orientation within the LBD	properly aligned toward the canonical agonist binding mode	shifted toward the H12 region, often causing steric interference
hydrophobic pocket occupation	balanced hydrophobic packing without conformational strain	dominant hydrophobic interactions leading to conformational constraint
overall conformational effect on VDR	supports activation-compatible receptor organization	inconsistent with an inactive-like receptor organization

Ligands that fail to engage His305 or that preferentially occupy space near H12 with bulky hydrophobic moieties tend to exhibit antagonistic behavior by restricting the H12 mobility or positioning.

### 3.5 Thermodynamic Analysis of Agonist and Antagonist Binding

MM-PBSA calculations were used to obtain a comparative energetic description of agonist and antagonist binding to *h*RARγ and *h*VDR. The resulting values of Δ*H*_eff_, −*T*Δ*S*_app_, and Δ*G*_bind_ for analyzed complexes are summarized in Tables 5 and 6, whereas group-level statistical comparisons are shown in Table 7 and Figure 8. Receptor-specific energetic distributions and the corresponding statistical summaries are provided in Figures S10 and S11. For the *h*RARγ series, the MM-PBSA results revealed a pronounced energetic distinction between agonist- and antagonist-bound complexes. Agonists consistently displayed more favorable Δ*G*_bind_ values than antagonists, with values ranging from −51.4 to −62.8 kcal mol^–1^ for agonists and from −36.8 to −50.0 kcal mol^–1^ for antagonists (Table 5). A similar pattern was observed for the enthalpic component, with agonists showing more favorable Δ*H*_eff_ values (−31.6 to −42.5 kcal mol^–1^) than antagonists (−16.1 to −28.6 kcal mol^–1^), whereas the estimated entropic contributions remained relatively constant (−*T*Δ*S*_app_ ≈ −20 kcal mol^–1^). In particular, CD1530 and palovarotene exhibited the most favorable Δ*G*_bind_ and Δ*H*_eff_ values among the *h*RARγ agonists, consistent with the formation of extensive stabilizing interactions within the ligand-binding pocket.

**Table 5:** MM-PBSA Binding Free Energies and Associated Effective Energetic Descriptors for *h*RARγ-Ligand Complexes.^a^

*h*RARγ - agonist	Δ*H*_eff_ [kcal mol^–1^]	–*T*Δ*S*_app_ [kcal mol^–1^]	Δ*G*_bind_ [kcal mol^–1^]
AGN205327	–37.5 ± 2.9	–20.2 ± 2.6	–57.7 ± 3.1
AGN204647	–33.4 ± 3.1	–20.2 ± 2.1	–53.5 ± 3.4
AGN191183 (pan-RAR)	–31.6 ± 3.0	–19.8 ± 2.1	–51.4 ± 3.1
CD1530	–42.5 ± 3.1	–20.3 ± 2.8	–62.8 ± 3.5
palovarotene	–39.9 ± 2.9	–20.4 ± 2.4	–60.3 ± 3.2

*h*RARγ - antagonist	Δ*H*_eff_ [kcal mol^–1^]	–*T*Δ*S*_app_ [kcal mol^–1^]	Δ*G*_bind_ [kcal mol^–1^]
AGN205728	–26.3 ± 3.5	–20.7 ± 2.8	–47.0 ± 2.3
AGN194310 (pan-RAR)	–16.1 ± 2.0	–20.7 ± 2.1	–36.8 ± 3.3
MM11253	–20.3 ± 2.8	–20.6 ± 2.5	–40.9 ± 3.0
LY2955303	–28.6 ± 2.9	–21.5 ± 2.1	–50.0 ± 2.8

a Binding free energies were calculated as Δ*G*_bind_ = *G*_complex_ – *G*_receptor_ – *G*_ligand_. The term Δ*H*_eff_ denotes an effective MM-PBSA energetic descriptor calculated as the sum of van der Waals, electrostatic, polar solvation, and nonpolar solvation contributions. Because the solvation terms are free-energy components, Δ*H*_eff_ should not be interpreted as a strict calorimetric enthalpy. The apparent residual entropic contribution was calculated as −*T*Δ*S*_app_ = Δ*G*_bind_ – Δ*H*_eff_ and is reported only as a qualitative descriptor. Values are reported as mean ± standard deviation (SD) across three independent MD replicas.

**Table 6:** MM-PBSA Binding Free Energies and Associated Effective Energetic Descriptors for *h*VDR-Ligand Complexes.^a^

*h*VDR - agonist	Δ*H*_eff_ [kcal mol^–1^]	–*T*Δ*S*_app_ [kcal mol^–1^]	Δ*G*_bind_ [kcal mol^–1^]
1,25D3	–32.0 ± 2.8	–20.5 ± 2.6	–52.5 ± 3.1
PRI-5202	–33.2 ± 2.5	–20.8 ± 1.8	–54.0 ± 3.8
PRI-1938	–37.5 ± 2.3	–20.8 ± 2.7	–58.3 ± 3.7

*h*VDR - antagonist	Δ*H*_eff_ [kcal mol^–1^]	–*T*Δ*S*_app_ [kcal mol^–1^]	Δ*G*_bind_[kcal mol^–1^]
ZK168281	–28.8 ± 3.0	–21.1 ± 2.9	–49.9 ± 3.6
ZK159222	–26.0 ± 3.7	–21.2 ± 3.3	–47.2 ± 3.7
ZK191784	–8.5 ± 3.1	–21.2 ± 2.5	–29.7 ± 3.3

a Binding free energies were calculated as Δ*G*_bind_ = *G*_complex_ – *G*_receptor_ – *G*_ligand_. The term Δ*H*_eff_ denotes an effective MM-PBSA energetic descriptor calculated as the sum of van der Waals, electrostatic, polar solvation, and nonpolar solvation contributions. Because the solvation terms are free-energy components, Δ*H*_eff_ should not be interpreted as a strict calorimetric enthalpy. The apparent residual entropic contribution was calculated as −*T*Δ*S*_app_ = Δ*G*_bind_ – Δ*H*_eff_ and is reported only as a qualitative descriptor. Values are reported as mean ± standard deviation (SD) across three independent MD replicas.

**Table 7:** Group-Level Statistical Comparison of MM-PBSA-Derived Energetic Descriptors between Agonist and Antagonist Complexes of *h*RARγ and *h*VDR.^a^

receptor	metric	agonists *n*	antagonists *n*	agonists (mean ± SD), kcal mol^–1^	antagonists (mean ± SD), kcal mol^–1^	Welch’s *t* test *p*-value
*h*RARγ	Δ*G*_bind_	5	4	–57.1 ± 4.7	–43.7 ± 5.9	0.011
*h*RARγ	Δ*H*_eff_	5	4	–37.0 ± 4.6	–22.8 ± 5.7	0.007
*h*VDR	Δ*G*_bind_	3	3	–54.9 ± 3.0	–42.3 ± 11.0	0.177
*h*VDR	Δ*H*_eff_	3	3	–34.2 ± 2.9	–21.1 ± 11.1	0.168

a Group means and standard deviations were calculated using one mean value per receptor–ligand complex. Statistical comparisons were performed using Welch’s two-tailed *t*-test.

**Figure 8:**
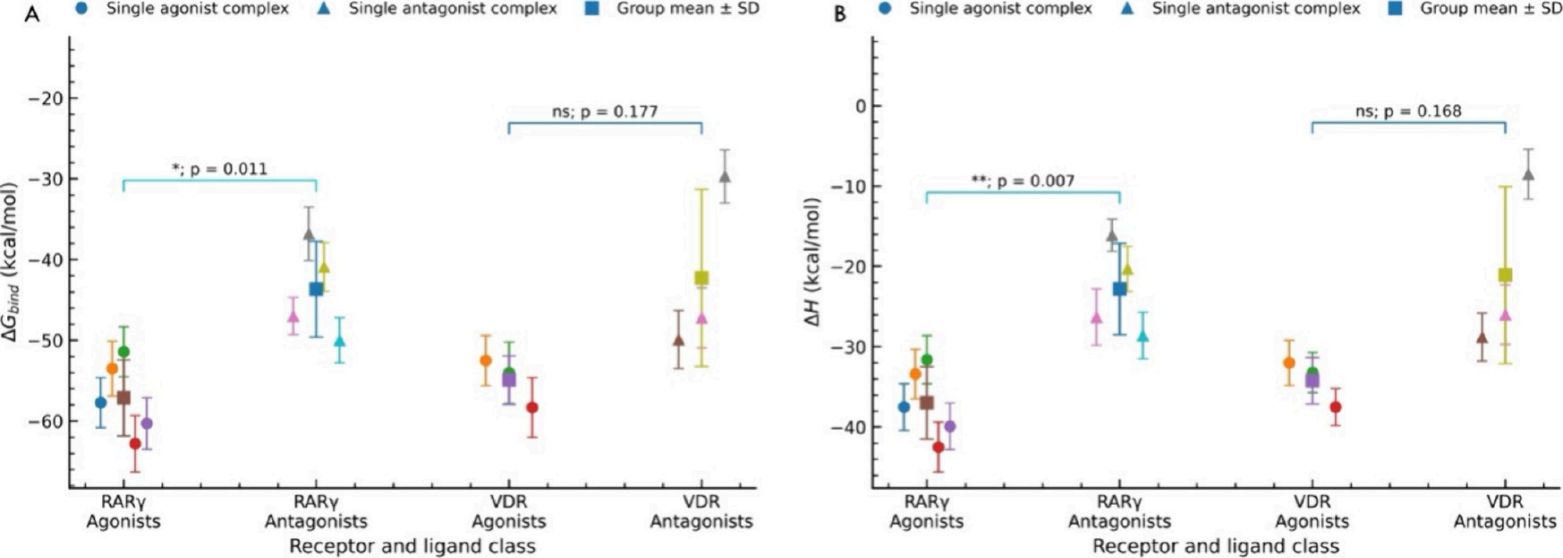
Group-level comparison of MM-PBSA-derived energetic descriptors for agonist and antagonist complexes of *h*RARγ and *h*VDR. (A) Δ*G*_bind_. (B) Δ*H*_eff_. Individual points represent mean values for individual receptor–ligand complexes; squares and error bars indicate group mean ± SD.

A similar directional trend was observed for *h*VDR ligands (Table 6), although the separation between agonists and antagonists was less pronounced than for *h*RARγ. Agonists showed more favorable Δ*G*_bind_ values (−52.5 to −58.3 kcal mol^–1^) than antagonists (−29.7 to −49.9 kcal mol^–1^) and likewise more favorable Δ*H*_eff_ values (−32.0 to −37.5 kcal mol^–1^ for agonists vs −8.5 to −28.8 kcal mol^–1^ for antagonists). The reduced separation in the *h*VDR series was accompanied by greater variability within the antagonist set, particularly due to the markedly less favorable energetic profile of ZK191784. At the same time, PRI-1938 combined strong enthalpic stabilization with increased H12 mobility (Section 3.6.3), indicating that favorable enthalpy alone is not sufficient to define a fully activation-competent conformational ensemble.

Thus, the energetic analysis should be interpreted together with the structural and dynamical data. Across both receptor systems, the MM-PBSA data indicate that calculated binding free energy alone is insufficient to unambiguously discriminate functional ligand classes, because some overlap in Δ*G*_bind_ remains between agonists and antagonists. However, the energetic results do reveal consistent comparative trends, particularly for *h*RARγ, where agonists are associated with more favorable effective enthalpic stabilization than antagonists. Importantly, because the apparent entropy term was obtained indirectly as a residual term from postprocessed trajectory data and was not subjected to rigorous convergence analysis, −*T*Δ*S*_app_ was not used here as a standalone mechanistic descriptor. Accordingly, −*T*Δ*S*_app_ should be regarded only as an internally consistent, purely qualitative descriptor and should therefore not be used for quantitative SAR comparisons or the rigorous formal free-energy decomposition analysis. Instead, the mechanistic interpretation is based primarily on Δ*G*_bind_, Δ*H*_eff_, and the accompanying structural and dynamical analyses.

To evaluate whether the energetic trends observed at the individual-complex level were statistically supported, group-level comparisons were performed using one mean value for each receptor–ligand complex. Agonist and antagonist subsets were compared separately for *h*RARγ and *h*VDR using Welch’s *t* test, and the results are summarized in Table 7 and shown in Figure 8. For the *h*RARγ series, agonists displayed significantly more favorable Δ*G*_bind_ and Δ*H*_eff_ values than antagonists. The mean Δ*G*_bind_ values were −57.1 ± 4.7 kcal mol^–1^ for agonists and −43.7 ± 5.9 kcal mol^–1^ for antagonists (*p* = 0.011), whereas the corresponding mean Δ*H*_eff_ values were −37.0 ± 4.6 and −22.8 ± 5.7 kcal mol^–1^ for agonists and antagonists, respectively (*p* = 0.007). These results indicate that, within the *h*RARγ data set, agonist and antagonist complexes are not only qualitatively differentiated but also statistically distinguishable at the group level in terms of their energetic profiles. For the *h*VDR series, the same directional trend was observed, with agonists showing more favorable mean Δ*G*_bind_ and Δ*H*_eff_ values than antagonists. However, these differences did not reach statistical significance within the present data set.

The mean Δ*G*_bind_ values were −54.9 ± 3.0 kcal mol^–1^ for agonists and −42.3 ± 11.0 kcal mol^–1^ for antagonists (*p* = 0.177), while the mean Δ*H*_eff_ values were −34.2 ± 2.9 and −21.1 ± 11.1 kcal mol^–1^, respectively (*p* = 0.168) (Table 7). Thus, the *h*VDR energetic pattern is directionally consistent with that observed for *h*RARγ, but it should be interpreted as an indicative, data set-specific trend rather than as a statistically resolved agonist–antagonist separation. The graphical summary in Figure 8 illustrates these group-level relationships.

In both panels, the *h*RARγ agonist and antagonist subsets are more clearly separated than the corresponding *h*VDR subsets. This difference is particularly evident for Δ*H*_eff_, supporting the interpretation that effective enthalpic stabilization is more closely associated with ligand-class separation in the *h*RARγ system. By contrast, the weaker separation in *h*VDR may reflect the relatively limited structural diversity of the analyzed agonists, together with greater heterogeneity within the antagonist subset. Under these conditions, ligand-class differences remain visible at the level of average energetic trends but are less readily resolved as statistically robust group-level separation.

To further quantify the descriptor-level separability suggested by the energetic distributions and group-level analysis, a retrospective threshold-based analysis was performed for Δ*G*_bind_ and Δ*H*_eff_ (Table 8). Within the present receptor-specific ligand panels, both descriptors showed an apparent internal separation of agonists and antagonists when thresholds were defined retrospectively using the full receptor-specific data set. This observation should be interpreted as an internal consistency result rather than as a statistically or externally validated predictive classification model. However, the margin and leave-one-ligand-out stability of this apparent internal separation differed between the two descriptors.

**Table 8:** Threshold-Based Internal Descriptor-Separability Analysis for Δ*G*_bind_ and Δ*H*_eff_.^a^

receptor	descriptor	boundary-defining pair	threshold [kcal mol^–1^]	minimum interclass gap [kcal mol^–1^]	apparent accuracy	leave-one-ligand-out accuracy
*h*RARγ	Δ*G*_bind_	AGN191183/LY2955303	–50.7	1.4	9/9, 100%	7/9, 77.8%
*h*RARγ	Δ*H*_eff_	AGN191183/LY2955303	–30.1	3.0	9/9, 100%	9/9, 100%
*h*VDR	Δ*G*_bind_	1,25D3/ZK168281	–51.2	2.6	6/6, 100%	5/6, 83.3%
*h*VDR	Δ*H*_eff_	1,25D3/ZK168281	–30.4	3.2	6/6, 100%	6/6, 100%

a The boundary-defining pair corresponds to the least favorable agonist and the antagonist with the most favorable, i.e., most negative, energetic descriptor value. These two ligands define the closest separation between the agonist and antagonist descriptor ranges. The threshold was calculated as the midpoint between these two values. The minimum interclass gap was calculated as *g* = *x*_ant,min_ – *x*_ag,max_, where *x*_ag,max_ is the least favorable agonist value and *x*_ant,min_ is the most favorable antagonist value for the analyzed descriptor. More negative descriptor values were assigned to the agonist class.

For *h*RARγ, Δ*G*_bind_ separated agonists and antagonists with a minimum interclass gap of 1.4 kcal mol^–1^, defined by AGN191183 as the least favorable agonist and LY2955303 as the most favorable antagonist. By contrast, Δ*H*_eff_ produced a wider interclass gap of 3.0 kcal mol^–1^ for the same receptor. A similar trend was observed for *h*VDR, for which the minimum interclass gap increased from 2.6 kcal mol^–1^ for Δ*G*_bind_ to 3.2 kcal mol^–1^ for Δ*H*_eff_. The leave-one-ligand-out threshold analysis further supported the greater stability of Δ*H*_eff_ as an internal descriptor of class-separability within the present data set. For *h*RARγ, Δ*G*_bind_ achieved a leave-one-ligand-out accuracy of 77.8%, whereas Δ*H*_eff_ retained 100% accuracy. For *h*VDR, the corresponding accuracies were 83.3% for Δ*G*_bind_ and 100% for Δ*H*_eff_. Thus, although Δ*G*_bind_ provides apparent class separation in the present data set, this separation is narrower and less stable than that obtained from Δ*H*_eff_. These results support the interpretation that ligand-class discrimination within the present data set is not fully captured by binding free energy alone. Instead, effective enthalpic stabilization, as reflected by Δ*H*_eff_, provides a more stable internal descriptor of agonist–antagonist separation and better complements the structural and dynamical analyses of ligand-dependent receptor activation.

Overall, the MM-PBSA results support a clearer energetic separation between agonists and antagonists in the *h*RARγ series than in the *h*VDR series. For *h*VDR, the same directional trend is present but remains less sharply resolved, suggesting a more modest and heterogeneous energetic response. The threshold-based descriptor-separability analysis further refines this conclusion by showing that although Δ*G*_bind_ provides apparent separation of agonist- and antagonist-bound complexes within the present ligand panels, this separation is narrower and less stable than that obtained from Δ*H*_eff_. Thus, within the present data set, Δ*H*_eff_ provided a more stable internal descriptor of ligand-class differences than Δ*G*_bind_ alone, particularly for *h*RARγ. For *h*VDR, the same pattern should be regarded as a directional trend rather than as statistically resolved separation. Therefore, the thermodynamic analysis is used here to describe comparative energetic trends rather than to provide standalone evidence for universal thermodynamic discrimination. In this framework, ligand-class differences are best interpreted through the combined analysis of Δ*G*_bind_, Δ*H*_eff_, and the accompanying structural and dynamical descriptors.

### 3.6 Ligand-Induced Helix 12 Dynamics as a Determinant of Function

#### 3.6.1 Global Stability of *h*RARγ and *h*VDR Complexes Assessed by RMSD

Backbone RMSD analysis was performed to assess the global structural stability of the simulated *h*RARγ and *h*VDR-ligand-binding domain systems during the production MD simulations (Figure 9). RMSD values were calculated relative to the protein backbone atoms of the equilibrated starting structures and were used exclusively as a global descriptor of conformational stability within the analyzed trajectories. For the *h*RARγ systems, both the apo receptor and ligand-bound complexes reached stable or quasi-stable RMSD plateaus after the initial relaxation phase. The apo *h*RARγ LBD stabilized after approximately 15–20 ns and fluctuated around ∼2.2 Å for the remainder of the trajectory. The ligand-bound systems displayed slightly higher but stable RMSD values. In particular, the *h*RARγ-AGN205728 complex fluctuated around ∼2.3–2.4 Å after equilibration, whereas the *h*RARγ-AGN205327 complex stabilized at approximately 2.4–2.5 Å. Importantly, no progressive increase in RMSD was observed during the later stages of the trajectories. This indicates that the overall RARγ LBD fold was preserved under the applied simulation conditions and that ligand binding did not induce global structural destabilization within the analyzed time scale.

**Figure 9:**
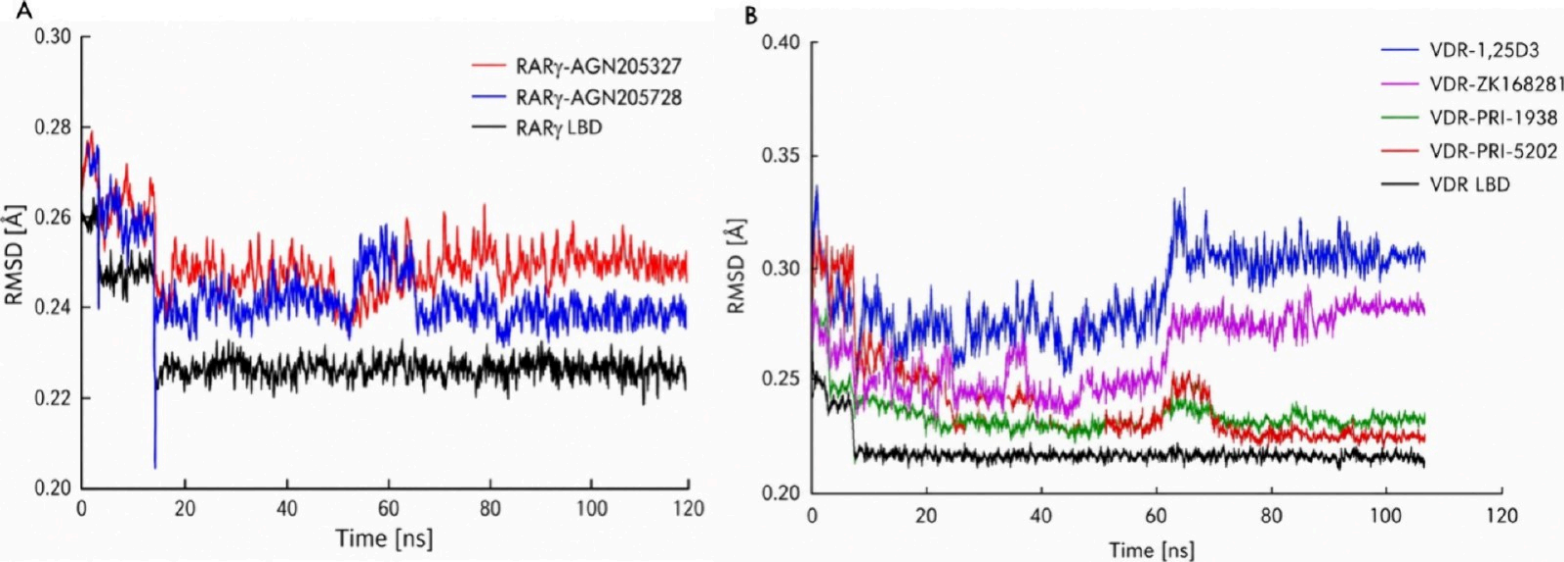
Backbone RMSD profiles of *h*RARγ and *h*VDR LBD systems during MD simulations. (A) RMSD profiles of apo and ligand-bound *h*RARγ LBD systems. (B) RMSD profiles of apo and ligand-bound *h*VDR LBD systems. RMSD values were calculated for the protein backbone atoms relative to the equilibrated starting structures. Representative traces are shown for clarity.

A comparable overall behavior was observed for the *h*VDR systems, although the magnitude and timing of RMSD stabilization differed between ligands. The apo *h*VDR LBD showed the lowest RMSD values, stabilizing at approximately ∼2.1–2.2 Å after the initial relaxation period. The PRI-1938 and PRI-5202 analogue complexes also maintained stable RMSD profiles, with *h*VDR-PRI-1938 fluctuating around ∼2.3–2.4 Å and *h*VDR-PRI-5202 remaining mostly within ∼2.3–2.5 Å. These values indicate that the PRI-bound *h*VDR complexes preserved the global LBD architecture during the analyzed production trajectories. In contrast, the *h*VDR-1,25D3 and *h*VDR-ZK168281 complexes exhibited ligand-dependent shifts toward higher RMSD plateaus. The *h*VDR-1,25D3 complex increased to approximately ∼3.0–3.1 Å after ∼60–65 ns, whereas *h*VDR-ZK168281 shifted to approximately ∼2.7–2.8 Å over a similar time interval. However, these increased RMSD values subsequently remained relatively stable and did not show continuous upward drift. Therefore, these changes are best interpreted as ligand-dependent conformational relaxation toward distinct metastable states rather than evidence of progressive unfolding or loss of structural integrity. This interpretation is also consistent with the preservation of the LBD fold throughout the trajectories and with the intrinsic flexibility expected of nuclear receptor LBDs, particularly in regions surrounding the H11/H12 and AF-2 structural elements.

Taken together, the RMSD profiles indicated that all simulated *h*RARγ and *h*VDR systems retained stable or quasi-stable global conformations during the production MD simulations. The observed RMSD differences between apo and ligand-bound states, as well as among individual ligand complexes, suggest ligand-specific conformational relaxation within the LBD rather than nonspecific destabilization. These results support the subsequent comparative analysis of residue-level flexibility, ligand–receptor interaction networks, H12/AF-2 organization, and MM-PBSA-derived energetic descriptors. Nevertheless, RMSD was used here primarily as a global stability metric and not as a standalone classifier of ligand efficacy or as evidence of complete conformational convergence, particularly with respect to large-scale H12/AF-2 rearrangements.

#### 3.6.2 Helix 12-Associated Dynamics in the *h*RARγ Ligand-Binding Domain

In NRs, ligand-dependent functional specificity is associated not only with the static geometry of ligand binding but also with the way ligand binding reorganizes local conformational dynamics within the LBD. Among the structural elements of the LBD, H12 is of particular importance in this context because it contributes directly to the AF-2 coactivator-binding surface and is structurally coupled to neighboring helices that stabilize the receptor activation interface. Crystallographic studies have shown that antagonist binding may displace H12 from the canonical agonist-like position without causing global disruption of the overall LBD fold.^53–55^ Therefore, analysis of H12-associated dynamics should consider not only the amplitude of H12 fluctuations but also H12 positioning, local stabilization, and the organization of the AF-2 regulatory surface.

To examine ligand-dependent flexibility in *h*RARγ, residue-resolved RMSF profiles were calculated for the LBD complexes bound to the agonist AGN205327 and the antagonist AGN205728 (Figure 10). RMSF values were obtained from the equilibrated portions of the MD trajectories. For each complex, three independent MD replica simulations were analyzed separately, followed by residue-wise averaging; inter-replica variability was reported as the standard deviation. This procedure minimizes the influence of trajectory-specific stochastic fluctuations and provides a more robust basis for comparing local dynamical trends between ligand-bound states.

**Figure 10:**
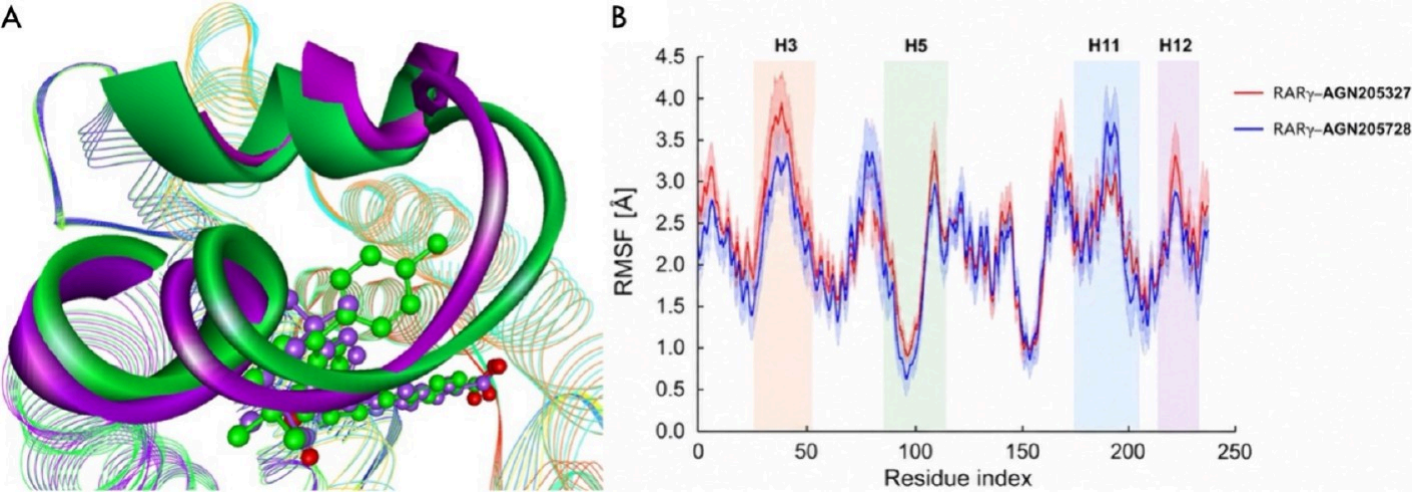
Ligand-dependent modulation of H12 dynamics in *h*RARγ complexes. (A) Superposition of MD-derived conformations from three independent replicas for *h*RARγ complexes with AGN205327 (agonist, magenta) and AGN205728 (antagonist, green). (B) Residue-level RMSF profiles averaged over three independent MD replicas for each system. Shaded areas indicate ± standard deviation (SD). Highlighted regions correspond to helices H3, H5, H11, and H12.

Both ligand-bound *h*RARγ systems preserved the overall LBD fold during the analyzed simulation window. Consistently, RMSF values averaged over the full LBD were nearly identical for the two complexes, reaching 2.39 ± 0.10 Å for AGN205327 and 2.41 ± 0.12 Å for AGN205728. These values indicate that antagonist binding was not associated with a global increase in receptor flexibility or nonspecific destabilization of the LBD scaffold. Instead, ligand-dependent differences were localized to selected structural regions linked to AF-2 organization. Thus, the differences observed in the RMSF profiles are more consistent with a localized redistribution of flexibility within functionally relevant regions of the LBD than with global structural disruption of the domain. A more detailed region-specific comparison revealed that the antagonist-bound complex displayed modestly elevated fluctuations in selected regions structurally coupled to the AF-2 environment (Table 9). The N-terminal segment of H3, spanning Leu224-Ala246, showed consistently higher RMSF values in the antagonist-bound state than in the agonist-bound state, with positive differential fluctuation (ΔRMSF) values of approximately +0.55 Å. A smaller but detectable increase in flexibility was also observed in the H5/loop region, Ala266-Thr287, with an average ΔRMSF of approximately +0.15 Å. Because these segments contribute to the structural framework supporting the AF-2 surface, their increased mobility suggests ligand-dependent modulation of local receptor dynamics beyond the immediate ligand-contacting residues. In contrast, H11 did not show a uniform directional response.

**Table 9:** Region-Specific RMSF Values for Selected Elements of the *h*RARγ LBD in Complexes with the Agonist AGN205327 and the Antagonist AGN205728.

		RMSF [Å]			
helix/region	representative residues	agonist	antagonist	^a^ΔRMSF [Å]	functional implication
H3 N-terminus	Leu224-Ala246	3.2 ± 0.20	3.75 ± 0.15	+0.55	modestly increased flexibility in the antagonist-bound state
H5/loop	Ala266-Thr287	3.4 ± 0.30	3.55 ± 0.45	+0.15	local flexibility increases in an H12-coupled region
H11	Ser390-Glu403	3.50 ± 0.50	3.45 ± 0.15	–0.05	mixed local behavior without a uniform trend
H12 (mean)	≈Pro410-Glu417	2.57 ± 0.15	2.60 ± 0.24	+0.03	very small difference between ligand-bound states

a ΔRMSF = RMSF_antagonist_ – RMSF_agonist_; positive values indicate higher flexibility in the antagonist-bound state. Values are reported as mean ± SD across three independent MD replicas.

Depending on the residue range considered, ΔRMSF values were either slightly positive or slightly negative, indicating heterogeneous local behavior rather than a consistent increase or decrease in flexibility. The mean RMSF of H12, defined here as Pro410–Glu417, was also nearly unchanged between the two systems, with values of 2.57 ± 0.15 Å for the agonist-bound complex and 2.60 ± 0.24 Å for the antagonist-bound complex. Thus, the RMSF data do not support a simplistic model in which antagonist binding globally increases the amplitude of H12 motion. This finding does not contradict the established mechanistic role of H12 in RARγ functional specificity. Rather, it indicates that the agonist–antagonist difference is not captured by H12 RMSF amplitude alone. In the complexes analyzed here, ligand-dependent behavior appears to involve a redistribution of flexibility across AF-2-associated structural elements, including the H3–H5–H11–H12 network surrounding the ligand-binding pocket and coactivator-binding surface. Therefore, H12 positioning, local ligand–receptor contacts, anchoring interactions, and AF-2 surface organization are likely more informative than the mean RMSF of H12 considered in isolation. These observations are consistent with a structural model in which antagonist-bound NR complexes may retain the overall LBD fold while altering the positioning or stabilization of H12 relative to the activation surface. In this context, the AGN205327-bound complex is more consistent with a compact and structurally coherent AF-2-associated environment, whereas the AGN205728-bound complex shows a modest redistribution of flexibility in neighboring anchoring and H12-coupled regions. Importantly, the observed RMSF differences are moderate and should not be interpreted as a direct quantitative measure of receptor activation, coactivator-binding competence, or long-range allosteric coupling. Instead, RMSF is used here as a comparative dynamical descriptor that complements the structural analysis of ligand positioning, H12 orientation, anchoring interactions, and helix-associated stabilization patterns. Overall, the RMSF results support ligand-dependent differences in the local conformational behavior of the RARγ LBD, with the principal distinction arising from localized AF-2-associated dynamical reorganization rather than from global receptor destabilization or a simple increase in H12 mobility.

#### 3.6.3 Helix 12 Modulation in *h*VDR Complexes

To extend the analysis of ligand-dependent H12-associated dynamics, residue-resolved RMSF profiles were calculated for the *h*VDR-ligand-binding domain in complex with the natural agonist 1,25D3, the antagonist ZK168281, and the agonist-like ligands PRI-5202 and PRI-1938 (Figure 11). The purpose of this analysis was not to assign pharmacological efficacy directly from RMSF values but to determine whether individual ligands produce distinct local mobility patterns within the H11–H12 region and other AF-2-associated elements of the LBD. For each complex, RMSF profiles were calculated independently from three MD replicas; residue-wise values were averaged across replicas, and inter-replica variability was reported as the standard deviation.

**Figure 11:**
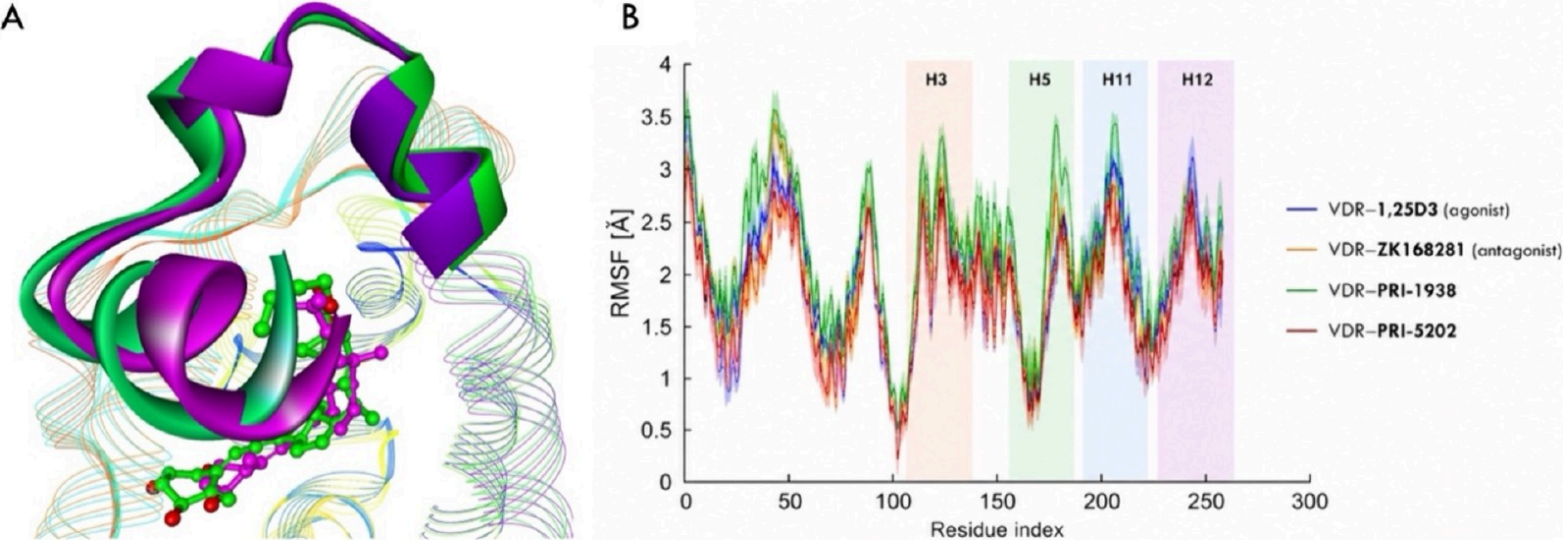
Ligand-dependent modulation of H12 dynamics in *h*VDR LBD complexes. (A) Superposition of MD-derived conformations from three independent replicas for *h*VDR bound to 1,25D3 (agonist, magenta) and ZK168281 (antagonist, green). (B) Residue-level RMSF averaged over three independent MD replicas for *h*VDR LBD complexes with 1,25D3, ZK168281, PRI-1938, and PRI-5202. Shaded areas indicate ± SD. Highlighted regions correspond to helices H3, H5, H11, and H12.

Across all *h*VDR systems, the overall LBD fold was retained during the analyzed trajectories, indicating that the observed RMSF differences reflect local dynamical effects rather than large-scale disruption of the receptor scaffold. Within this preserved structural framework, the ligands produced distinct mobility profiles, most notably in the H11–H12 segment and at selected residues involved in ligand anchoring and AF-2-associated packing. The natural agonist 1,25D3 showed a comparatively restrained RMSF profile in the H11–H12 region, consistent with maintenance of a compact agonist-associated arrangement of the C-terminal part of the LBD. A similar pattern was observed for PRI-5202, whose RMSF values remained close to those of 1,25D3 at several key positions, including Ser237, Arg274, and His305 (Table 10). Among the analyzed synthetic ligands, PRI-5202 therefore most closely reproduced the local mobility pattern of the natural agonist, particularly in regions linked to H12 stabilization and AF-2 organization. PRI-1938 displayed a heterogeneous dynamical profile. Although this ligand retained several contacts compatible with agonist-like binding, its RMSF values were elevated at multiple AF-2-associated positions. The mean RMSF calculated for H12, defined here as Cys410-Leu417, increased to 2.5 ± 0.10 Å for PRI-1938, compared with 2.3 ± 0.15 Å for 1,25D3 and 2.2 ± 0.10 Å for PRI-5202. This difference was also evident at Glu409, located at the H11–H12/AF-2 boundary, where the RMSF for PRI-1938 reached 3.2 ± 0.20 Å and exceeded the values observed for both 1,25D3 and PRI-5202 (Figure 11B and Table 10).

**Table 10:** Region-Specific RMSF Values for Selected Residues and Structural Elements of the *h*VDR-Ligand-Binding Domain in Complexes with 1,25D3, ZK168281, PRI-1938, and PRI-5202.^b^

		RMSF [Å]				^a^ΔRMSF [Å]	
region/residue	structural role	1,25D3	ZK168281	PRI-1938	PRI-5202	PRI-1938	PRI-5202
Ser237 (H3)	ligand anchor	1.0 ± 0.05	0.9 ± 0.15	1.2 ± 0.05	0.9 ± 0.05	+0.20	–0.11
Arg274 (H5)	polar contact	0.6 ± 0.05	0.7 ± 0.11	0.9 ± 0.05	0.7 ± 0.05	+0.30	+0.03
His305 (loop H6–H7)	central stabilization	1.7 ± 0.10	1.9 ± 0.10	2.0 ± 0.11	1.6 ± 0.15	+0.30	–0.06
His397 (H11)	pre-AF-2 packing	1.3 ± 0.10	1.5 ± 0.15	1.6 ± 0.10	1.4 ± 0.10	+0.30	+0.08
H12 average (Cys410-Leu417)	AF-2 helix	2.3 ± 0.15	2.2 ± 0.20	2.5 ± 0.10	2.1 ± 0.10	+0.20	–0.18
H12 (Glu409)	C-terminal H12 flexibility	3.1 ± 0.20	3.1 ± 0.20	3.2 ± 0.20	2.8 ± 0.20	+0.10	–0.25

a ΔRMSF = RMSF_ligand_ – RMSF_1,25D3_. Values are reported as mean ± SD across three independent MD replicas.

b RMSF values are reported as mean ± SD across three independent MD replicas.

Increased fluctuations were also observed at residues contributing to ligand anchoring or local packing including Ser237 and His305. These results indicate that the preservation of selected agonist-like contacts does not necessarily imply the same degree of local dynamical restraint as observed in the 1,25D3 and PRI-5202 complexes. The antagonist ZK168281 also altered the *h*VDR RMSF profile relative to that of 1,25D3, but its effect was not characterized by a uniform increase in mobility across the entire AF-2-associated region. Instead, the antagonist-bound complex showed a ligand-specific redistribution of fluctuations, supporting the view that *h*VDR conformational behavior is better described by a spatial distribution of residue-level mobility than by a single average RMSF value.

ΔRMSF analysis relative to the 1,25D3 reference complex further highlighted these ligand-dependent differences. PRI-5202 showed near-zero or slightly negative ΔRMSF values at several H11–H12-associated positions, consistent with a mobility profile close to that of the natural agonist. In contrast, PRI-1938 displayed positive ΔRMSF values at Ser237, Arg274, His305, His397, and the averaged H12 region, indicating a broader and less uniformly restrained local conformational ensemble. Thus, although PRI-1938 maintains several ligand–receptor interactions associated with agonist-like binding, these interactions are accompanied by weaker local stabilization of the AF-2/H12 region than observed for 1,25D3 or PRI-5202. These results suggest that static ligand–receptor contacts alone are insufficient to describe ligand-dependent conformational behavior in *h*VDR. The analyzed ligands differ not only in their binding interactions but also in how these interactions are propagated into local mobility changes within the H11–H12/AF-2 region. In this respect, PRI-5202 remains closest to the natural agonist reference state, whereas PRI-1938 is associated with enhanced local flexibility at selected AF-2-associated positions. ZK168281 likewise perturbs the local mobility pattern, although not through a simple global mobilization of H12. The RMSF profiles should not be interpreted as evidence that agonist-like ligands universally suppress H12 motion, whereas antagonist-like ligands uniformly enhance it. Rather, the data indicate that *h*VDR ligands modulate residue-level flexibility in a region-specific manner across structurally coupled elements surrounding the ligand-binding pocket and the AF-2 surface. Accordingly, the spatial distribution of mobility across H12 and neighboring anchoring elements provides a more informative descriptor of ligand-dependent local dynamics than the absolute RMSF of H12 considered in isolation. The RMSF analysis does not directly quantify receptor activation, coactivator-binding competence, or activation-competent conformational sampling. Here, it is therefore used as a comparative descriptor of local dynamics rather than as standalone evidence of agonism, antagonism, or partial agonism. Integrated with the analysis of ligand positioning, anchoring interactions, H12 orientation, and local stabilization patterns, the RMSF profiles reveal ligand-dependent differences in the conformational behavior of the *h*VDR LBD. Specifically, 1,25D3 and PRI-5202 maintain a more restrained and structurally coherent AF-2-related environment, whereas PRI-1938 and ZK168281 produce more pronounced local perturbations within selected elements of this region. Nevertheless, the present 100 ns conventional MD simulations should be interpreted as providing comparisons of local relaxation, H12-adjacent packing, ligand-dependent stability, and short-time scale H12-related trends rather than as exhaustive sampling of large-scale H12 reorientation or complete activation-inactivation transitions. Longer simulations and enhanced sampling approaches, such as accelerated MD or metadynamics, would therefore be valuable to further validate the observed H12-associated trends.

## 4 Conclusions

In this study, we present an integrated structural and computational framework combining molecular docking with the GOLD using the ChemPLP scoring function, MD simulations, and MM-PBSA-derived energetic descriptors to compare molecular determinants associated with agonism and antagonism in the *h*RARγ and *h*VDR nuclear receptor LBDs. The protocol reproduced reference-binding poses in redocking tests and enabled an internally consistent comparative analysis of ligand-binding modes, supporting its use for structure-based interpretation of ligand function within structurally conserved ligand-binding domains. A central observation of this work is that the ligand functional class is associated with the subtle modulation of the interaction network controlling H12 and the AF-2 region, rather than with large structural rearrangements of the ligand-binding domain within the time scale sampled here. In the *h*VDR system, 1,25D3 and the agonist-like ligands PRI-5202 and PRI-1938 maintain, to different extents, activation-compatible interaction patterns involving His305, Ser237, and Arg274, together with H12-compatible ligand positioning and packing. In contrast, ZK168281, ZK159222, and ZK191784 show incomplete or geometrically less productive His305-associated engagement and favor H12-proximal hydrophobic interactions involving residues such as Leu414, Val418, and Phe422, consistent with restricted or destabilized H12 packing. In the *h*RARγ system, antagonism correlates with loss of Arg278 anchoring interactions and increased engagement of the posterior binding pocket, providing a structural basis for agonist-to-antagonist functional switching. Dynamic analyses further suggest that functional selectivity is associated with ligand-dependent redistribution of local flexibility within the AF-2 regulatory region, rather than with global destabilization of the receptor scaffold. While global RMSF profiles remain largely conserved across complexes, agonists preferentially maintain H12-compatible packing, whereas antagonists perturb local H12-associated interaction patterns within the H3–H5–H11–H12 regulatory region. The observation that analogue local dynamic signatures emerged for both *h*RARγ and *h*VDR suggests that modulation of H12-associated dynamics may represent a recurrent mechanistic feature within the present ligand set, although its generality will require validation using additional ligands and longer or enhanced sampling simulations.

Importantly, the thermodynamic analysis should be interpreted in light of the limitations of the applied MM-PBSA workflow. The apparent entropic term, −*T*Δ*S*_app_, was derived indirectly as Δ*G*_bind_ – Δ*H*_eff_ and was therefore treated only as a qualitative, internally consistent descriptor rather than as a rigorous configurational entropy estimate. It should not be used for quantitative SAR comparisons or rigorous formal free-energy decomposition analyses. Within the present MM-PBSA-based analysis, the clearest thermodynamic distinction between agonist- and antagonist-bound complexes was observed for *h*RARγ, where agonists showed more favorable Δ*G*_bind_ and Δ*H*_eff_ values. For *h*VDR, the same energetic pattern was observed only as a directional trend and was not statistically resolved within the present data set. Therefore, the thermodynamic results are best interpreted as comparative energetic trends that complement the structural and dynamical analyses, rather than as standalone evidence for universal thermodynamic discrimination.

An additional distinctive aspect of this work is the integration of MicroED-based nanoscale crystallography with computational modeling. MicroED enabled de novo determination of the structures of challenging RARγ ligands, AGN205728 and AGN194310, from submicron crystals, providing atomic-level information on their solid-state conformations and hydrogen-bonding motifs. These experimentally determined ligand structures provide useful conformational references for modeling, but they should not be interpreted as constituting direct evidence of receptor-bound bioactive conformations, particularly in the presence of possible polymorphic or crystal-packing effects. Taken together, these results support the use of docking, MD simulations, MM-PBSA-derived effective energetic descriptors, and MicroED-derived structural data as a complementary strategy for dissecting ligand-dependent receptor behavior.

The present work should be regarded as a retrospective mechanistic benchmark rather than a rigorously validated predictive framework. Future studies incorporating longer simulations, enhanced sampling approaches, rigorous entropy estimation, multidimensional analyses of collective motions and correlated dynamics, and independent external ligand sets will be required to determine whether the proposed structural, energetic, and H12-related descriptors can be generalized for predictive classification or rational design of selective *h*RARγ and *h*VDR modulators.

## Supplementary Material



## Data Availability

All data supporting the findings of this study are available within the article and its Supporting Information. Representative MD-equilibrated receptor–ligand structures are provided to illustrate key binding modes, H12-adjacent packing, and ligand–residue interaction patterns. These structures correspond to snapshots selected from equilibrated portions of production trajectories.

## Notes

Structural templates used for docking; docking validation results; ESP-fitting quality metrics for ligand partial charges; MicroED crystal data and refinement statistics for AGN194310 and AGN205728; ligand–receptor interactions identified from docking models and MD simulations; quantitative MD-derived structural descriptors for *h*RARγ and *h*VDR; molecular packing diagrams; redocking validation results; docking versus MD binding mode comparisons; two-dimensional ligand–receptor interaction schematics; receptor-specific MM-PBSA energetic comparisons; representative MD-equilibrated receptor–ligand PDB structures (PDF)
