# Supplementary material for: Structural and Thermodynamic Discrimination between Agonists and Antagonists of Retinoic Acid Receptor γ and the Vitamin D Receptor
Source: J Chem Inf Model. 2026 Jun 30;66(13):7701–21. doi: 10.1021/acs.jcim.6c00912 (PMC13370873; doi:10.1021/acs.jcim.6c00912)
Supplement: Supplementary file 1 [file ci6c00912_si_001.pdf]

Supporting Information

Structural and Thermodynamic Discrimination Between Agonists and Antagonists of RARγ and VDR Nuclear Receptors

Teresa Żołek[1,], Szymon Sutuła[2], Andrzej Kutner[3]*

[1]Department of Organic and Physical Chemistry, Faculty of Pharmacy, Medical University of Warsaw, 1 Banacha, 02-097 Warsaw, Poland

[2]Cryomicroscopy and Electron Diffraction Core Facility, Centre of New Technologies, University of Warsaw, 2c Banacha, 02-097 Warsaw, Poland

[3]Department of Drug Chemistry, Pharmaceutical and Biomedical Analysis, Faculty of Pharmacy, Medical University of Warsaw, 1 Banacha, 02-097 Warsaw, Poland

List of Tables

Table S1. Structural templates used for docking.

Table S2. Docking validation results for the *h*RARγ and *h*VDR systems used for docking protocol selection. Re-docking was performed for crystallographic agonist complexes, whereas AGN205728 was included as an antagonist control because no antagonist-bound *h*RARγ crystal structure is available. Docked poses were compared with the corresponding reference ligand conformations where available. RMSD values are reported in Å, and scores were used for pose ranking within each receptor-specific docking system.

Table S3. Electrostatic potential fitting quality metrics for ligand partial charges derived using the ChelpG scheme in Gaussian 16. The table reports the total molecular charge, spin

multiplicity, number of ESP fitting points, RMS and relative RMS error of the ESP fit, and the sum of fitted ESP charges for each ligand.

Table S4. MicroED crystal data and structure refinement for AGN194310.

Table S5. MicroED crystal data and structure refinement for AGN205728.

Table S6. Ligand-*h*RARγ interactions identified from docking models and MD simulations.

Table S7. Quantitative MD-derived structural descriptors supporting the *h*RARγ interaction features and their relationship to class-level MM-PBSA trends.

Table S8. Ligand-*h*VDR interactions identified from docking models and MD simulations.

Table S9. Quantitative MD-derived structural descriptors supporting the *h*VDR interaction features and their relationship to class-level MM-PBSA trends.

List of Figures

Figure S1. Re-docking validation for the *h*RARγ and *h*VDR docking templates. Superposition of crystallographic (blue) and ChemPLP re-docked (green) poses of (A) CD564 in *h*RARγ LBD (PDB ID: 1FCY; RMSD = 0.30 Å) and (B) 1,25D3 in *h*VDR LBD (PDB ID: 1DB1; RMSD = 1.38 Å). The crystallographic poses were used as references for defining the ligand-binding pockets and validating pose recovery.

Figure S2. Molecular packing of AGN194310 viewed along the *a* axis with hydrogen atoms omitted, shown in stick representation.

Figure S3. The comparison of B–A–B molecular chains in the AGN205728 α- and β-forms.

Figure S4. Molecular packing of the AGN205728 α-form (A) and β-form (B) viewed along the *a* axis, with hydrogen atoms omitted, shown in stick representation.

Figure S5. Quantitative MD-derived structural descriptors supporting receptor-specific agonist/antagonist interaction patterns. (A) *h*RARγ ligand–residue structural descriptors for agonist- and antagonist-bound complexes, shown as mean ± SD for the representative contact distances listed in Table S7. (B) *h*VDR ligand–residue structural descriptors for agonist/agonist-like and antagonist-bound complexes, shown as mean ± SD for the representative contact distances listed in Table S9. For both panels, labels above the data points indicate contact occurrence as the number of ligands exhibiting a given interaction relative to the total number of ligands in the corresponding class. Contacts classified as absent were not plotted as distance values. The H12-core centroid descriptor represents the distance between the ligand-centroid and the H12-associated core region. For *h*VDR His305, the agonist-side value corresponds to the direct hydrogen bond observed for 1,25D3 only, whereas PRI-5202 and PRI-1938 showed hydrophobic proximity rather than a direct His305 hydrogen bond.

Figure S6. Comparison of AGN205728 binding modes obtained by molecular docking and MD simulation. (A) Overlay of α-AGN205728 (blue) and β-AGN205728 (orange) in the *h*RARγ

ligand-bonding pocket based on the docking pose generated with GOLD. (B) Representative post-MD binding mode of α-AGN205728 (blue) and β-AGN205728 (orange) after MD simulation, showing the equilibrated ligand arrangement.

Figure S7. The two-dimensional representations of *h*RARγ and agonist and pan-RAR agonist interactions.

Figure S8. The two-dimensional schematic representations of *h*RARγ and the antagonist and pan-RAR antagonist interactions.

Figure S9. The two-dimensional representations of *h*VDR and agonist (1,25D3, PRI-5202 and PRI-1938) and antagonist (ZK168281, ZK159222, and ZK191784) interactions.

Figure S10. Receptor-specific comparison of MM-PBSA-derived (A) binding free energies G_{bind} and (B) enthalpic contributions ΔH_{eff} for *h*RARγ agonist and antagonist complexes**.** Individual points represent mean values for single receptor-ligand complexes, labeled by ligand name. Squares and error bars indicate group mean ± SD. The bracket indicates the result of the group-level statistical comparison.

Figure S11. Receptor-specific comparison of MM-PBSA-derived (A) binding free energies $G_{\mathrm{bind}}$and (B) enthalpic contributions ΔH_{eff} for *h*VDR agonist and antagonist complexes**.** Individual points represent mean values for single receptor-ligand complexes, labeled by ligand name. Squares and error bars indicate group mean ± SD. The bracket indicates the result of the group-level statistical comparison.

Table S1. Structural templates used for docking.

Receptor	PDB ID	Ligand	Functional state	Resolution	Mutation status	Modeled-region completeness	Rationale
*h*RARγ LBD	1FCY	CD564	Agonist-bound	1.30 Å	No reported mutations	Complete deposited LBD construct	High-resolution human RARγ agonist-bound template defining the canonical LBP
*h*VDR LBD	1DB1	1,25D3	Agonist-bound	1.80 Å	No reported mutations	Nearly complete modeled LBD construct	High-resolution human VDR agonist-bound template with natural ligand

Note: The *h*VDR structure corresponds to an LBD construct lacking the variable *h*VDR-specific insertion domain; however, this construct has been reported to retain ligand binding and transcriptional activity comparable to the wild-type receptor.

Table S2. Docking validation results for the *h*RARγ and *h*VDR systems used for docking protocol selection. Re-docking was performed for crystallographic agonist complexes, whereas AGN205728 was included as an antagonist control because no antagonist-bound *h*RARγ crystal structure is available. Docked poses were compared with the corresponding reference ligand conformations where available. RMSD values are reported in Å, and scores were used for pose ranking within each receptor-specific docking system.

Receptor	Reference ligand	Scoring function	Docking experiment	Score	RMSD (Å)
*h*RARγ	CD564 (agonist)	ChemPLP	Re-docking of crystallographic ligand	120.16	0.30
*h*RARγ	CD564 (agonist)	GOLDScore		101.36	0.30
*h*RARγ	CD564 (agonist)	ChemScore		62.54	0.40
*h*RARγ	CD564 (agonist)	ASP		53.60	0.30
*h*RARγ	AGN205728 (antagonist)	ChemPLP	Antagonist control docking	58.26	7.28
*h*RARγ	AGN205728 (antagonist)	GOLDScore		4.45	7.07
*h*RARγ	AGN205728 (antagonist)	ChemScore		39.62	6.74
*h*RARγ	AGN205728 (antagonist)	ASP		19.17	6.71
*h*VDR	1,25D3 (agonist)	ChemPLP	Re-docking of crystallographic ligand	69.29	1.38
*h*VDR	1,25D3 (agonist)	GOLDScore		65.63	1.35
*h*VDR	1,25D3 (agonist)	ChemScore		42.48	1.63
*h*VDR	1,25D3 (agonist)	ASP		41.11	9.54

Table S3. Electrostatic potential fitting quality metrics for ligand partial charges derived using the CHelpG scheme in Gaussian 16. The table reports the total molecular charge, spin multiplicity, number of ESP fitting points, RMS and relative RMS error of the ESP fit, and the sum of fitted ESP charges for each ligand.

Ligand	Charge	Multiplicity	QM method	Charge model	ESP fit points	ESP RMS	ESP RRMS	Sum of ESP charges
AGN205327					2026	0.00144	0.09736	0.00000
AGN204647					1962	0.00144	0.10311	0.00000
AGN191183 (pan-RAR)					1866	0.0012	0.11354	0.00000
CD1530					1888	0.00087	0.07719	0.00000
palovarotene					2241	0.00154	0.09987	0.00000
AGN205327					2026	0.00144	0.09736	0.00000
AGN205728					2352	0.00146	0.14294	0.00000
AGN194310 (pan-RAR)	0	1	B3LYP/6-311G++(d,p)	ESP	2332	0.00137	0.13318	0.00000
MM11253					2191	0.00178	0.13396	0.00000
LY2955303					2982	0.00137	0.09610	0.00000
1,25D3					2368	0.00121	0.11790	0.00000
PRI-5202					2559	0.00132	0.12777	0.00000
PRI-1938					2477	0.00117	0.10477	0.00000
ZK168281					2758	0.00129	0.11679	0.00000
ZK159222					2776	0.00125	0.10359	0.00000
ZK191784					2684	0.00149	0.11707	0.00000

Table S4. MicroED crystal data and structure refinement for AGN194310.

Empirical formula	$C_{112}H_{96}O_8S_4$
Formula weight	1698.26
Temperature/K	80.00
Crystal system	monoclinic
Space group	$P2_1/c$
a/Å	5.6(2)
b/Å	34.8(3)
c/Å	25.4(2)
α/°	90
β/°	91.74(16)
γ/°	90
Volume/Å^3	4939(193)
Z	2
ρ_{calc} g/cm^3	1.142
Crystal size/mm^3	0.001 × 0.0002 × 0.0002
Radiation	electron (λ = 0.0251)
2Θ range for data collection/°	0.136 to 1.652
Index ranges	$-6 \leq h \leq 6$, $-39 \leq k \leq 40$, $-29 \leq l \leq 29$
Reflections collected	35929
Independent reflections	7808 [R_{int} = 0.5045, R_{sigma} = 0.3433]
Data/restraints/parameters	7808/79/524
Goodness-of-fit on F^2	0.970
Final R indexes [I>=2σ (I)]	R_1 = 0.1532, wR_2 = 0.3765
Final R indexes [all data]	R_1 = 0.3015, wR_2 = 0.4571

Table S5. MicroED crystal data and structure refinement for AGN205728.

	AGN205728 α-form	AGN205728 β-form
Empirical formula	$C_{29}H_{27}NO_3$	$C_{29}H_{27}NO_3$
Formula weight	437.54	437.54
Temperature/K	80.00	80.00
Crystal system	triclinic	triclinic
Space group	P-1	P-1
a/Å	8.5270(7)	8.7516(5)
b/Å	14.3362)	15.2488(12)
c/Å	19.583(3)	18.7658(11)
α/°	86.045(11)	89.052(6)
β/°	83.042(8)	82.059(5)
γ/°	80.635(9)	80.476(6)
Volume/Å^3	2341.7(5)	2446.1(3)
Z	4	4
ρ_{calc} g/cm^3	1.241	1.188
Radiation	electron (λ = 0.0251)	electron (λ = 0.0251)
2Θ range for data collection/°	0.122 to 1.712	0.122 to 1.718
Index ranges	$-10 \le h \le 10$, $-17 \le k \le 17$, $-23 \le l \le 23$	$-10 \le h \le 10$, $-15 \le k \le 15$, $-22 \le l \le 22$
Reflections collected	28579	20237
Independent reflections	8277 [R_{int} = 0.2960, R_{sigma} = 0.2374]	6885 [R_{int} = 0.1603, R_{sigma} = 0.1497]
Data/restraints/parameters	8277/93/758	6885/5/614
Goodness-of-fit on F^2	1.095	1.159
Final R indexes [I>=2σ (I)]	R_1 = 0.1583, wR_2 = 0.3826	R_1 = 0.1446, wR_2 = 0.3690
Final R indexes [all data]	R_1 = 0.2763, wR_2 = 0.4554	R_1 = 0.2357, wR_2 = 0.4282

Table S6. Ligand-*h*RARγ interactions identified from docking models and MD simulations.

Residue	*h*RARγ - ligand								
	Agonist					Antagonist			
	AGN 205327	AGN 204647	palovarotene	CD 1530	AGN 191183	AGN 205728	MM 11253	LY 2955303	AGN 194310
Arg278	HB	HB	HB	HB	-	-	-	-	-
Ser289	HB	HB	HB	HB	-	HB	HB	Carbon HB	HB
Phe288	Carbon HB π-alkyl	Carbon HB, π-alkyl	Carbon HB	Carbon HB	π-π stacked, π-alkyl	π-π stacked	Carbon HB, π-alkyl	Carbon HB, π-alkyl	Carbon HB, π-alkyl
Phe304	π-π stacked	π-alkyl	π-π stacked, π-alkyl	π-π stacked	π-π stacked, π-alkyl	π-π stacked, π-alkyl	π-alkyl	π-π stacked, π-alkyl	π-alkyl
Phe230	π-π stacked	π-π stacked	π-π stacked, π-alkyl	-	π-π stacked, π-alkyl	π-π stacked, π-alkyl	π-π stacked, π-alkyl	π-π stacked, π-alkyl	π-alkyl
Met272	alkyl	alkyl	alkyl	alkyl	alkyl	Carbon HB	alkyl	π-sulfur	alkyl
Leu268	alkyl	alkyl	alkyl	alkyl	alkyl	alkyl	-	-	alkyl
Leu233	-	-	-	HB	π-π stacked, alkyl	-	-	-	-
Leu271	HB, alkyl	alkyl	alkyl	alkyl, π-sigma	alkyl	HB, alkyl	alkyl	alkyl, π-sigma	alkyl
Leu307	π-π stacked	alkyl	alkyl	-	alkyl	alkyl	alkyl	-	-
Ala234	alkyl	alkyl	alkyl	alkyl	alkyl	alkyl	alkyl	alkyl	alkyl
Cys237	alkyl, π-sulfur	π-sulfur	HB, alkyl	alkyl	π-sulfur	alkyl	Carbon HB, alkyl	π-sulfur	π-sulfur
Ile275	alkyl	alkyl	alkyl	alkyl	alkyl	alkyl	alkyl	alkyl	alkyl
Gly393	-	-	-	-	-		-	Carbon HB	-
Gly303	-	-	-	-	-	-	-	Carbon HB	-
Arg396	alkyl, π-alkyl	-	alkyl, π-alkyl	alkyl, π-alkyl	alkyl, π-alkyl	alkyl, π-alkyl	alkyl, π-alkyl	Carbon HB	alkyl, π-alkyl
Ala397	alkyl	alkyl	alkyl	alkyl	alkyl	alkyl	alkyl	alkyl	alkyl
Trp227	alkyl, π-alkyl	alkyl, π-alkyl	alkyl, π-alkyl	-	alkyl, π-alkyl	alkyl, π-alkyl	alkyl, π-alkyl	-	π-π stacked, π-alkyl
Leu400	alkyl	-	alkyl	alkyl	alkyl	alkyl	alkyl	alkyl	alkyl
Met408	alkyl	alkyl	-	-	alkyl	alkyl, π-sulfur	alkyl	alkyl	-
Pro409	-	-	-	-	-	alkyl	-	-	alkyl
Ile412	alkyl	alkyl	alkyl	alkyl	alkyl	alkyl	alkyl	alkyl	alkyl
Met415	alkyl	-	alkyl	-	alkyl	-	alkyl	alkyl	-
Leu416	alkyl	alkyl	alkyl	-	alkyl	-	alkyl	alkyl	-
Met299	-	-	π-sulfur	-	-	-	-	-	-
Met286	-	-	π-sulfur	-	-	-	-	-	-
Ile389	-	-	-	-	-	-	-	alkyl	-
TIP3P	-	-	-	-	-	HB	-	-	HB
Val311	-	-	-	-	-	-	-	alkyl, π-alkyl	-

Table S7. Quantitative MD-derived structural descriptors supporting the *h*RARγ interaction features and their relationship to class-level MM-PBSA trends.

Feature/ residue	Interaction	Descriptor	Agonists, mean ± SD, [Å]	Antagonists, mean ± SD, [Å]	Interpretation
Arg278	H-bond	closest donor–acceptor distance	2.00 ± 0.20 (4/5)	absent (0/4)	Agonist-specific polar anchor supporting anterior ligand orientation.
Ser289	H-bond	closest donor–acceptor distance	2.06 ± 0.39 (4/5)	2.04 ± 0.21 (4/4)	Conserved secondary polar anchoring across ligand classes.
Phe304	π–π / π–alkyl / hydrophobic contact	closest aromatic/heavy-atom contact distance	3.95 ± 0.42 (5/5)	3.6 ± 0.12 (4/4)	Conserved aromatic/hydrophobic interaction; increased variability in agonists reflects multiple posterior binding modes, whereas in antagonists this interaction stabilizes posterior ligand positioning.
Leu400	posterior hydrophobic pocket contact	closest heavy-atom distance	5.14 ± 0.15 (4/5)	5.23 ± 0.17 (4/4)	More consistently observed in antagonists, consistent with enhanced posterior pocket engagement.
Met408	posterior/H11-adjacent hydrophobic contact	closest heavy-atom distance	4.62 ± 0.20 (3/5)	4.27 ± 0.48 (3/4)	Antagonists show a slightly shorter and more frequent Met408 contact, consistent with a tendency toward deeper posterior ligand positioning.
Pro409	hydrophobic contact	closest heavy-atom distance	Absent (0/5)	4.04 ± 0.18 (2/4)	Antagonist-specific interaction indicating ligand penetration toward the H11/H12 region.
Ile412	H12-adjacent hydrophobic contact	closest heavy-atom distance	4.07 ± 0.47 (5/5)	4.07 ± 0.81 (4/4)	Conserved contact, but increased variability in antagonists suggests altered H12-adjacent packing.
Met415	H12-adjacent hydrophobic contact	closest heavy-atom distance	5.03 ± 0.27 (3/5)	4.63± 0.15 (2/4)	Reduced antagonist occurrence suggests weaker or less conserved H12-adjacent packing.
Leu416	H12-adjacent hydrophobic contact	closest heavy-atom distance	4.75 ± 0.50 (4/5)	4.61 ± 0.69 (2/4)	Partly conserved but less frequent antagonist-associated H12-adjacent interactions.
H12-associated ligand position	ligand positioning near H12/core region	ligand-centroid distance to H12-associated/core region	7.04 ± 0.45 (5/5)	6.43 ± 0.13 (4/4)	Antagonists show shorter ligand-centroid distances to the H12-associated region, consistent with deeper posterior engagement and altered H12-adjacent packing.

Note: Values were calculated from ligand-residue interaction distances identified in docking and MD-refined interaction analyses. For each ligand-residue pair, the shortest observed distance was used as a representative descriptor, as it captures the most relevant interaction event, including transient but functionally significant

contacts. For the H12-core ligand-centroid descriptor, ligand-level averages were first computed when multiple values were available and then used to calculate class-level mean ± standard deviation. The reported mean ± SD values reflect results aggregated across three independent MD replicas. Contact occurrence is reported in parentheses as the number of ligands exhibiting the interaction relative to the total number of ligands within the functional class. This approach provides quantitative structural descriptors that enable comparison of interaction patterns across ligand classes without implying formal residue-wise energetic decomposition.

Table S8. Ligand-*h*VDR interactions identified from docking models and MD simulations.

Residue	*h*VDR - ligand					
	Agonist			Antagonist		
	1,25D3	PRI-5202	PRI-1938	ZK191784	ZK159222	ZK168281
His305	HB	alkyl, π-alkyl	alkyl, π-alkyl	Carbon HB	Carbon HB	-
Ser237	HB	HB	HB	-	HB	HB
Arg274	Carbon HB	Carbon HB	HB	-	HB	HB
Leu227	-	alkyl	alkyl	alkyl	alkyl	-
Ile271	-	-	-	alkyl	-	alkyl
Met272	-	alkyl, π-alkyl	alkyl, π-alkyl	alkyl, π-alkyl	alkyl, π-alkyl	alkyl, π-alkyl
Ser275	-	-	Carbon HB	Carbon HB	-	Carbon HB
Ser278	-	HB	HB	-	-	HB
Leu230	-	-	-	alkyl, π-alkyl	-	alkyl, π-alkyl
Ala231	alkyl, π-alkyl	-	alkyl, π-alkyl	-	alkyl, π-alkyl	-
Leu233	-	alkyl, π-alkyl	alkyl, π-alkyl	alkyl, π-alkyl	alkyl, π-alkyl	alkyl, π-alkyl
Val234	alkyl, π-alkyl	alkyl, π-alkyl	alkyl, π-alkyl	alkyl, π-alkyl	-	alkyl, π-alkyl
Trp286	alkyl, π-alkyl	alkyl, π-alkyl	alkyl, π-alkyl	alkyl, π-alkyl	alkyl, π-alkyl	alkyl, π-alkyl
Ile268	-	alkyl, π-alkyl	alkyl, π-alkyl	alkyl, π-alkyl	-	-
Cys288	HB π-alkyl	alkyl, π-alkyl	alkyl, π-alkyl	alkyl, π-alkyl	-	alkyl, π-alkyl
Tyr295	alkyl, π-alkyl	-	-	alkyl, π-alkyl	alkyl, π-alkyl	-
Tyr143	-	HB	HB	alkyl, π-alkyl	-	HB
Tyr147	-	-	alkyl, π-alkyl	alkyl, π-alkyl	-	-
Phe150	-	-	alkyl, π-alkyl	-	-	-
Val300	alkyl, π-alkyl	alkyl, π-alkyl	alkyl, π-alkyl	alkyl, π-alkyl	-	-
Ala303	alkyl, π-alkyl	HB	alkyl, π-alkyl	Carbon HB	-	alkyl, π-alkyl
Leu309	alkyl, π-alkyl	alkyl, π-alkyl	alkyl, π-alkyl	-	-	alkyl, π-alkyl
Leu313	-	-	alkyl, π-alkyl	-	alkyl, π-alkyl	alkyl, π-alkyl
Leu320	alkyl, π-alkyl	-	alkyl, π-alkyl	-	-	-
His397	alkyl, π-alkyl	alkyl, π-alkyl	alkyl, π-alkyl	Carbon HB	-	HB
Tyr401	alkyl, π-alkyl	alkyl, π-alkyl	alkyl, π-alkyl	-	-	-
Leu414	-	alkyl, π-alkyl	alkyl, π-alkyl	-	alkyl, π-alkyl	alkyl, π-alkyl
Leu417	-	-	-	-	-	-
Val418	alkyl,	alkyl,	alkyl,	alkyl,	alkyl,	alkyl,

	π-alkyl	π-alkyl	π-alkyl	π-alkyl	π-alkyl	π-alkyl
Leu419	alkyl, π-alkyl	-	-	-	-	-
Phe422	-	alkyl, π-alkyl	alkyl, π-alkyl	alkyl, π-alkyl	-	-

Table S9. Quantitative MD-derived structural descriptors supporting the *h*VDR interaction features and their relationship to class-level MM-PBSA trends.

Feature/residue	Interaction	Descriptor	*h*VDR agonists/agonist-like ligands, mean ± SD, [Å]	*h*VDR antagonists, mean ± SD, [Å]	Interpretation
His305	Activation-switch polar/proximity contact	Closest donor–acceptor distance for direct H-bond; hydrophobic proximity noted separately where no H-bond is present	2.14, direct H-bond in 1,25D3 only; PRI-5202 and PRI-1938 show hydrophobic proximity rather than direct H-bond	2.54 ± 0.63 (2/3)	Direct His305 hydrogen bonding is clearly retained in the reference agonist 1,25D3, but His305 alone should not be treated as an exclusive class marker. Functional interpretation should focus on the complete His305-Ser237-Arg274 network and H12-compatible ligand positioning.
Ser237	Ligand-anchoring H-bond	Closest donor–acceptor distance	1.60 ± 0.37 (3/3)	1.88 ± 0.08 (2/3)	Ser237 acts as a conserved polar anchoring residue. Its presence in both classes indicates that it is supportive but not independently discriminative.
Arg274	Polar network/ligand anchoring	Closest donor–acceptor distance	1.78 ± 0.16 (3/3)	1.80 ± 0.06 (2/3)	Arg274 participates in the polar anchoring network in both classes. The discriminating feature is not Arg274 alone, but whether the His305–Ser237-Arg274 network remains geometrically compatible with an active VDR conformation.
Tyr401	H12-adjacent hydrophobic contact	Closest heavy-atom distance	4.99 ± 0.12 3/3	absent 0/3	Tyr401 contacts are preferentially associated with agonist/agonist-like binding modes and may support H12-compatible packing.
Leu414	H12-adjacent hydrophobic contact	Closest heavy-atom distance	5.12 ± 0.30 (2/3)	4.75 ± 0.01 (2/3)	Shorter Leu414 contacts in antagonists support increased H12-adjacent hydrophobic packing, consistent with antagonist-associated steric constraint near H12.
Val418	H12-adjacent hydrophobic contact	Closest heavy-atom distance	4.47 ± 0.21 (3/3)	4.16 ± 0.50 (3/3)	Val418 contacts are conserved in both classes, but shorter antagonist

					distances suggest tighter H12-adjacent packing and possible steric restriction of H12 mobility.
Phe422	Posterior/H12-adjacent hydrophobic contact	Closest heavy-atom distance	4.87 ± 0.04 (2/3)	4.79 ± 0.04 (1/3)	Phe422 should be interpreted as ligand-specific rather than a general antagonist marker in the present dataset.
H12-associated ligand position	Ligand positioning near H12/core region	Ligand-centroid distance to the H12-associated/core region	7.84 ± 0.67, (3/3)	6.78 ± 0.15 (3/3)	Antagonists show shorter ligand-centroid distances to the H12-associated region, supporting a shifted H12-proximal binding mode and altered H12-adjacent packing.

Note: Values were derived from ligand-residue contacts identified in docking models and MD-refined conformations. For residue-level descriptors, the shortest observed ligand-residue distance was used as a representative contact descriptor for each ligand. Class-level values are reported as mean ± SD across ligands exhibiting the contact, with contact occurrence shown in parentheses. For the H12-associated ligand-position descriptor, ligand-level centroid distances were first averaged for each ligand and then used to calculate class-level mean ± SD. These descriptors should be interpreted as quantitative structural correlates of the global MM-PBSA trends, not as formal residue-wise MM-PBSA energetic contributions.

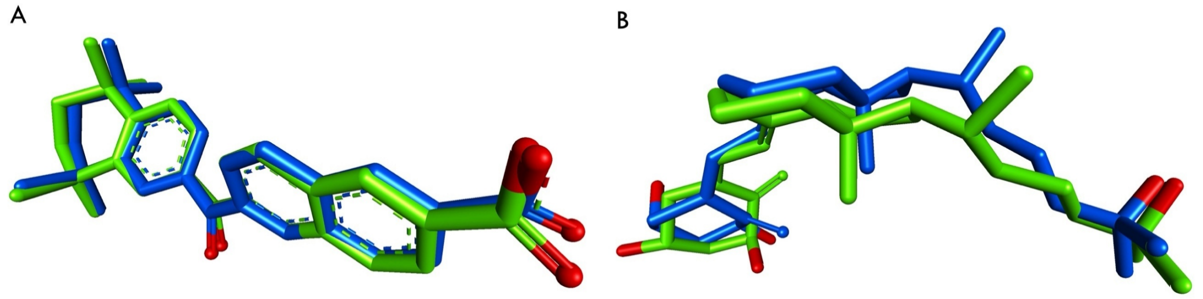

Figure S1. Re-docking validation for the *h*RARγ and *h*VDR docking templates. Superposition of crystallographic (blue) and ChemPLP re-docked (green) poses of (A) CD564 in *h*RARγ LBD (PDB ID: 1FCY; RMSD = 0.30 Å) and (B) 1,25D3 in *h*VDR LBD (PDB ID: 1DB1; RMSD = 1.38 Å). The crystallographic poses were used as references for defining the ligand-binding pockets and validating pose recovery.

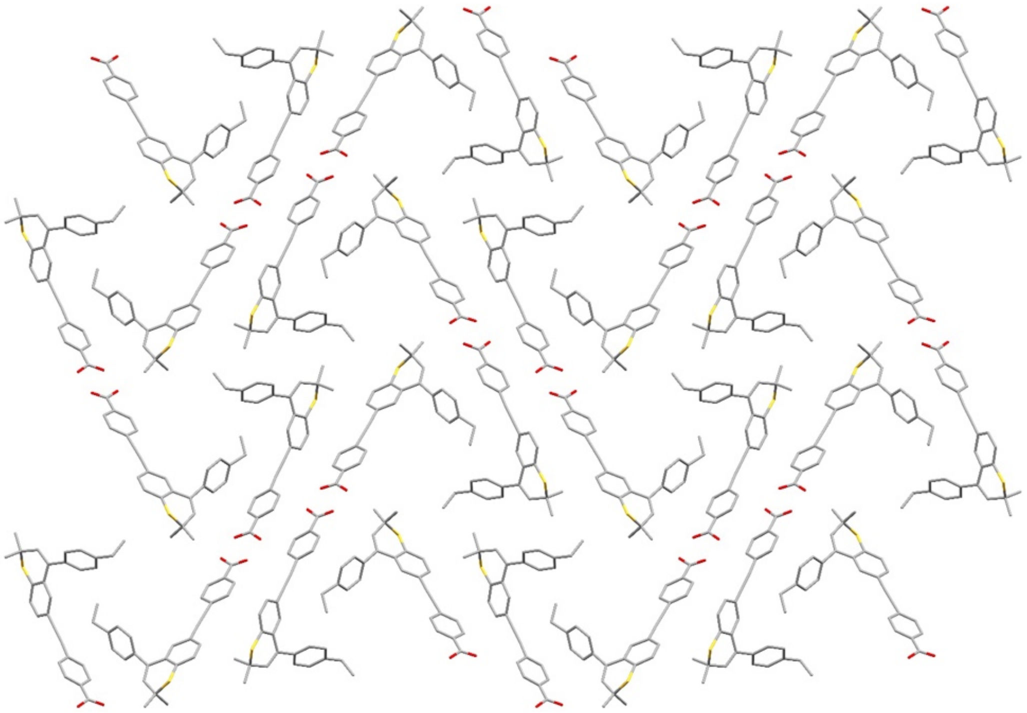

Figure S2. Molecular packing of AGN194310 viewed along the *a* axis with hydrogen atoms omitted, shown in stick representation.

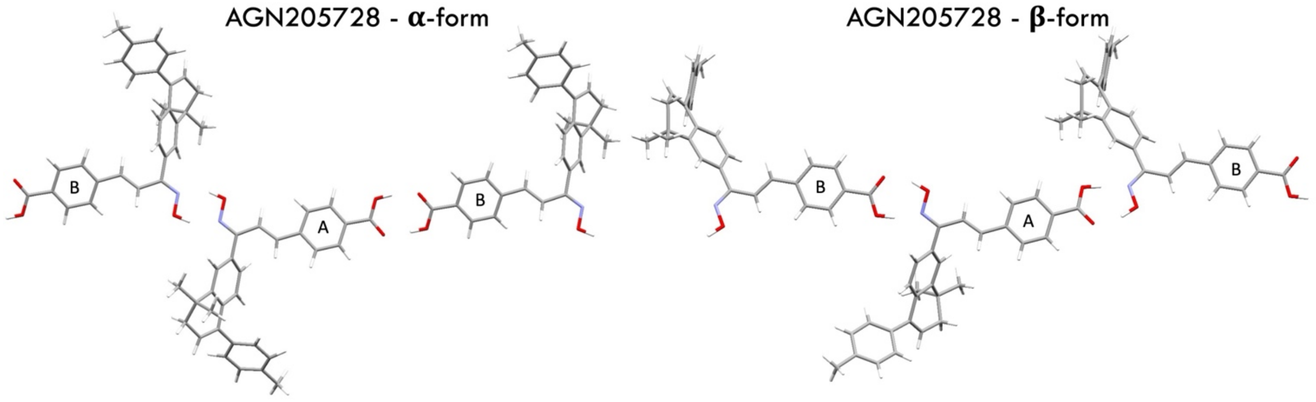

Figure S3. The comparison of B–A–B molecular chains in the AGN205728 α- and β-forms.

A

B

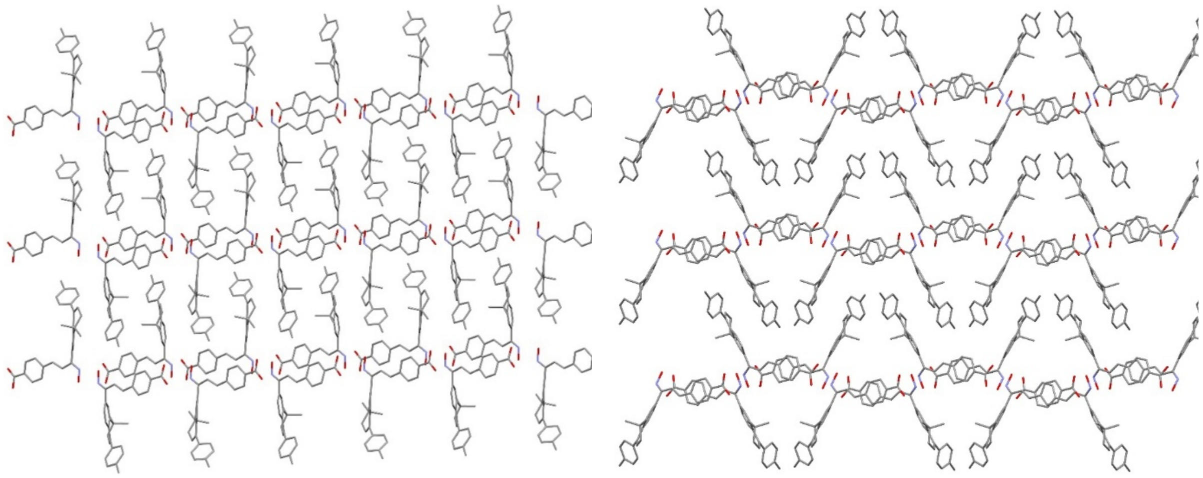

Figure S4. Molecular packing of the AGN205728 α-form (A) and β-form (B) viewed along an *a*-axis, with hydrogen atoms omitted, shown in stick representation.

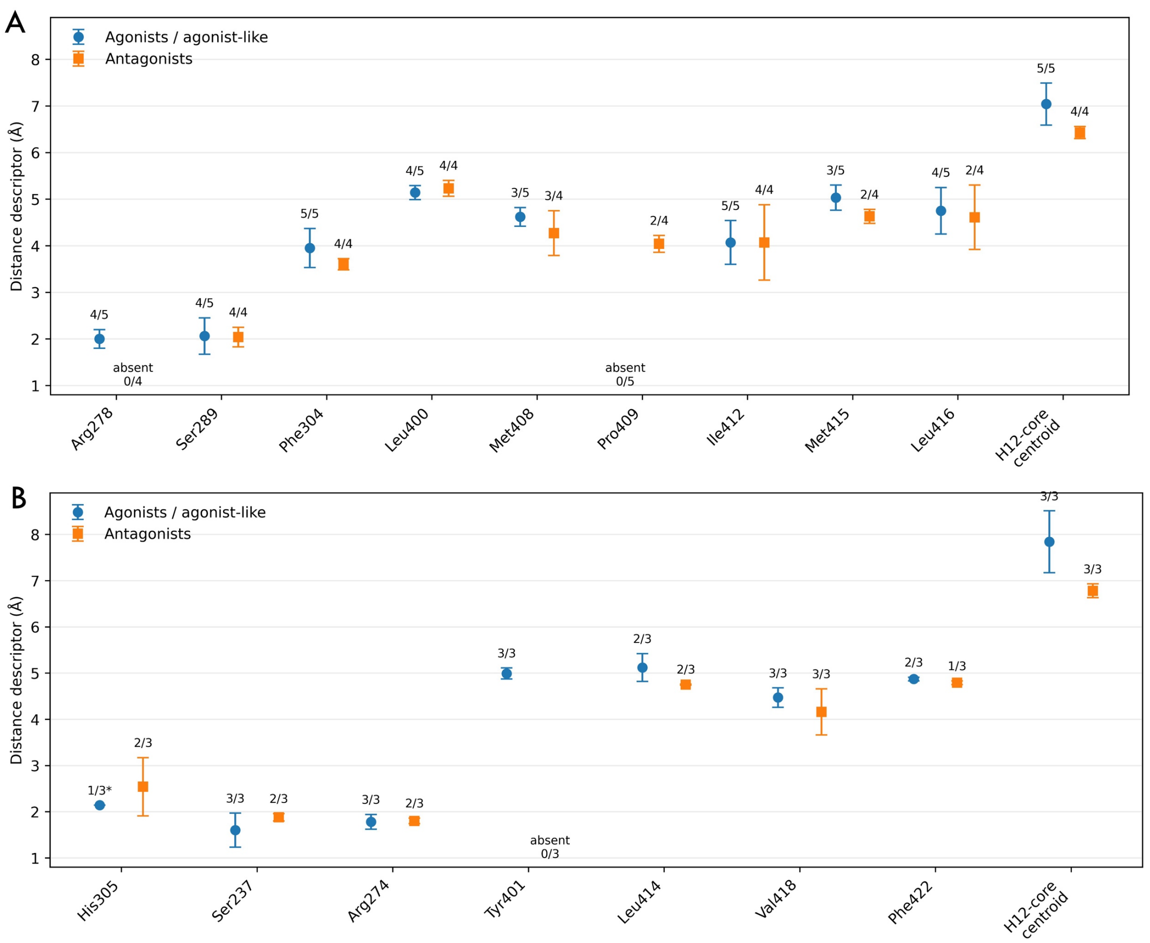

Figure S5. Quantitative MD-derived structural descriptors supporting receptor-specific agonist/antagonist interaction patterns. (A) *h*RARγ ligand-residue structural descriptors for agonist- and antagonist-bound complexes, shown as mean ± SD for the representative contact distances listed in Table S7. (B) *h*VDR ligand-residue structural descriptors for agonist/agonist-like and antagonist-bound complexes, shown as mean ± SD for the representative contact distances listed in Table S9. For both panels, labels above the data points indicate contact occurrence as the number of ligands exhibiting a given interaction relative to the total number of ligands in the corresponding class. Contacts classified as absent were not plotted as distance values. The H12-core centroid descriptor represents the distance between the ligand-centroid and the H12-associated core region. For *h*VDR His305, the agonist-side value corresponds to the direct hydrogen bond observed for 1,25D3 only, whereas PRI-5202 and PRI-1938 showed hydrophobic proximity rather than a direct His305 hydrogen bond.

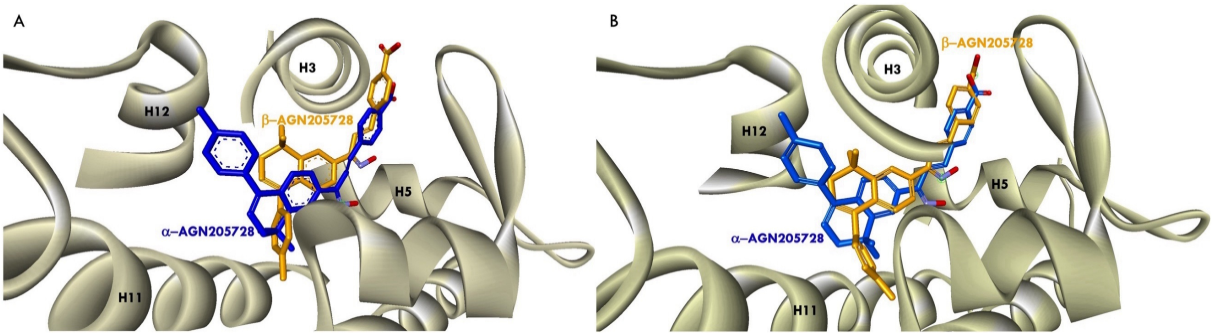

Figure S6. Comparison of AGN205728 binding modes from molecular docking and molecular dynamics (MD) simulations. (A) Overlay of α-AGN205728 (blue) and β-AGN205728 (orange) in the *h*RARγ ligand-binding pocket based on docking poses generated with GOLD. (B) MD-derived binding modes of α-AGN205728 (blue) and β-AGN205728 (orange), showing equilibrated ligand conformations.

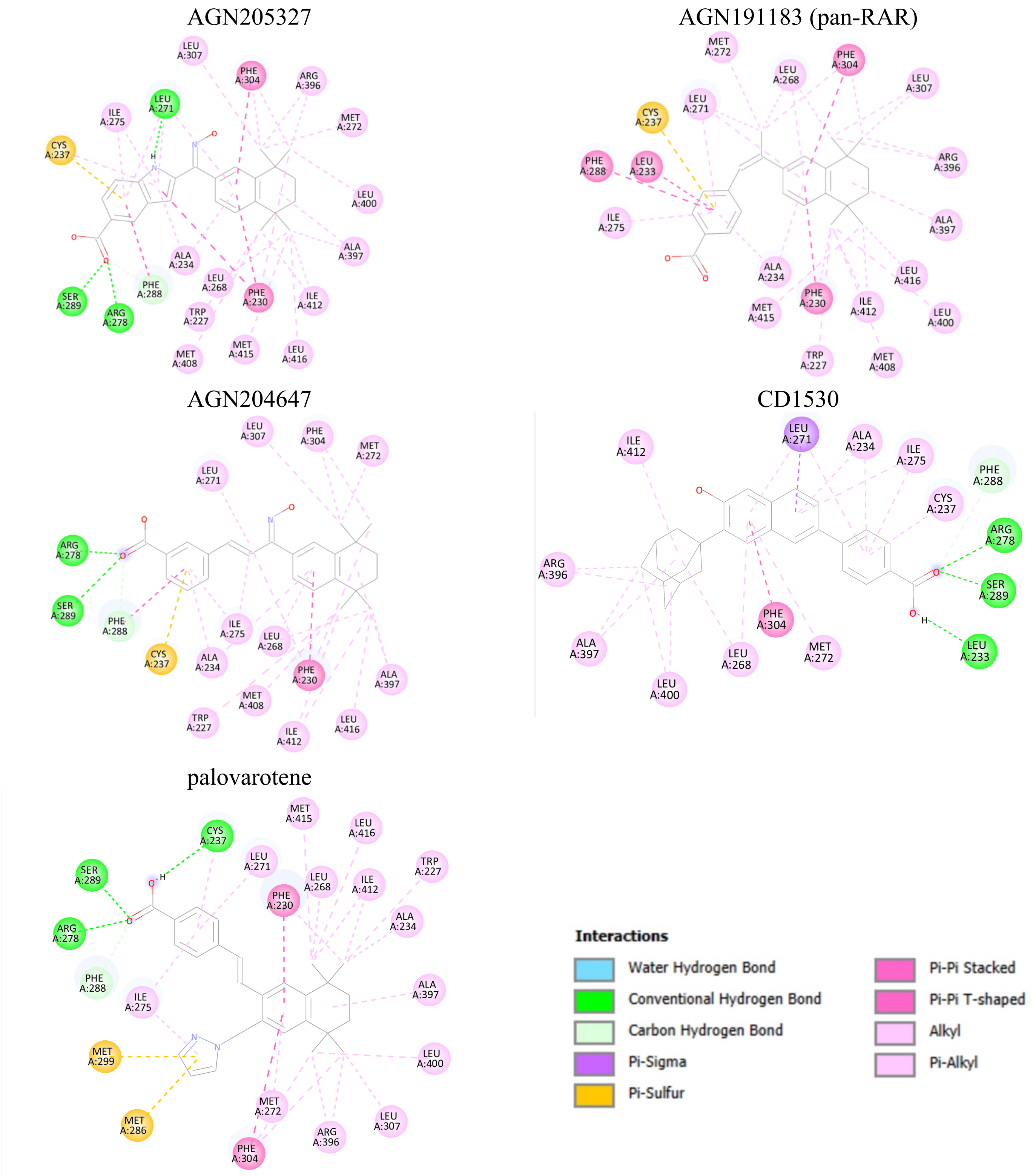

Figure S7. The two-dimensional representations of *h*RARγ and agonist and pan-RAR agonist interactions.

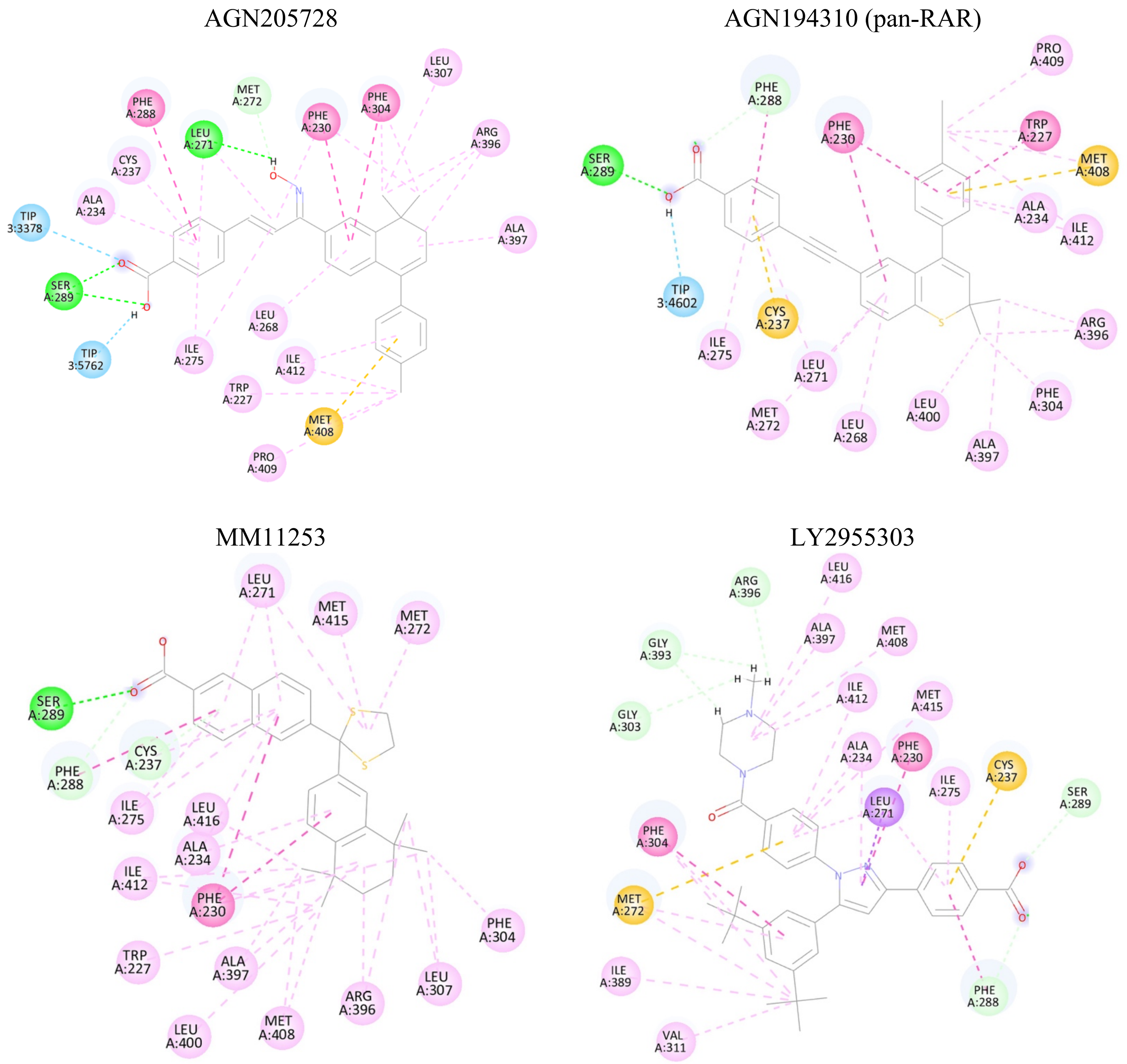

Figure S8. The two-dimensional schematic representations of *h*RARγ and the antagonist and pan-RAR antagonist interactions.

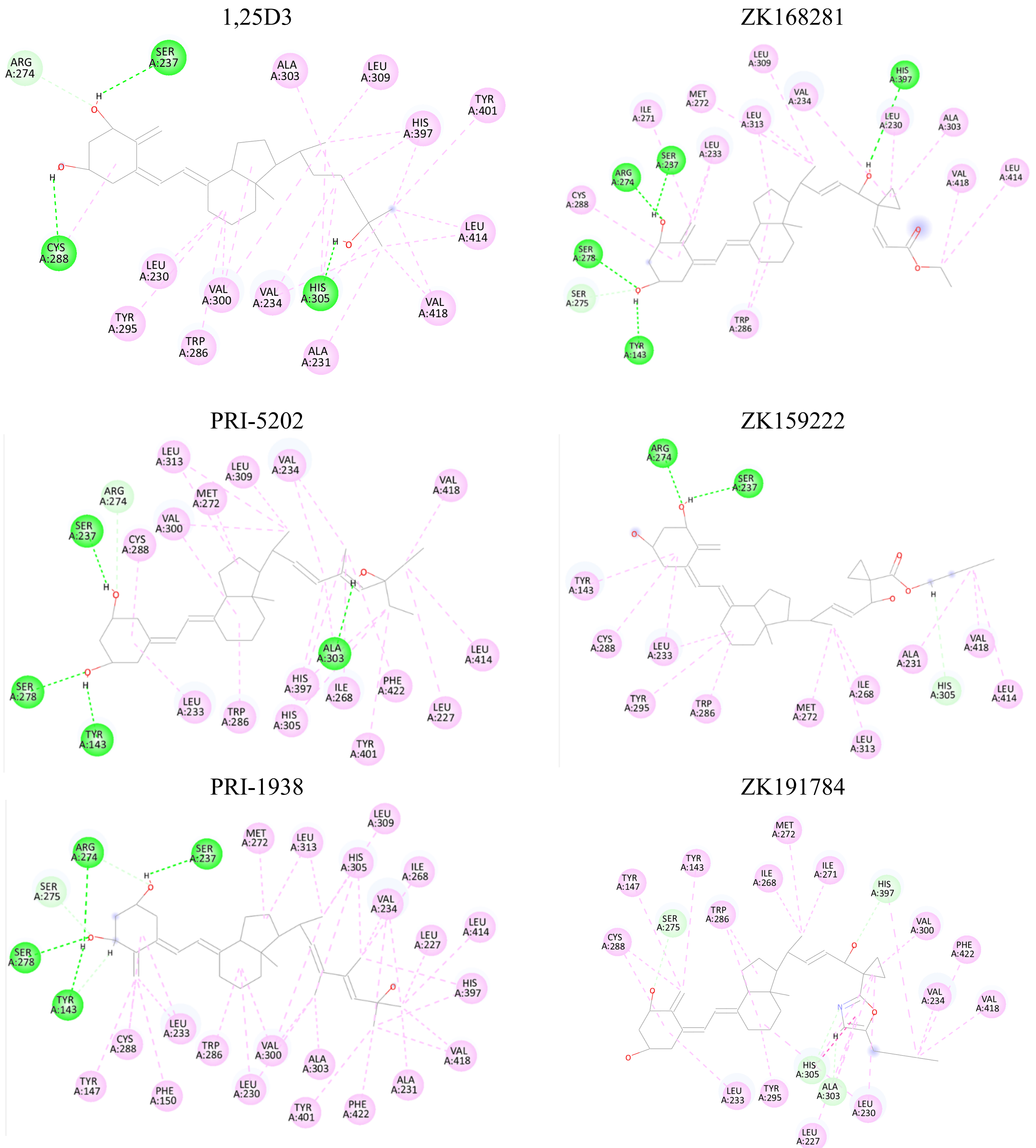

Figure S9. The two-dimensional representations of *h*VDR and agonist (1,25D3, PRI-5202 and PRI-1938) and antagonist (ZK168281, ZK159222, and ZK191784) interactions.

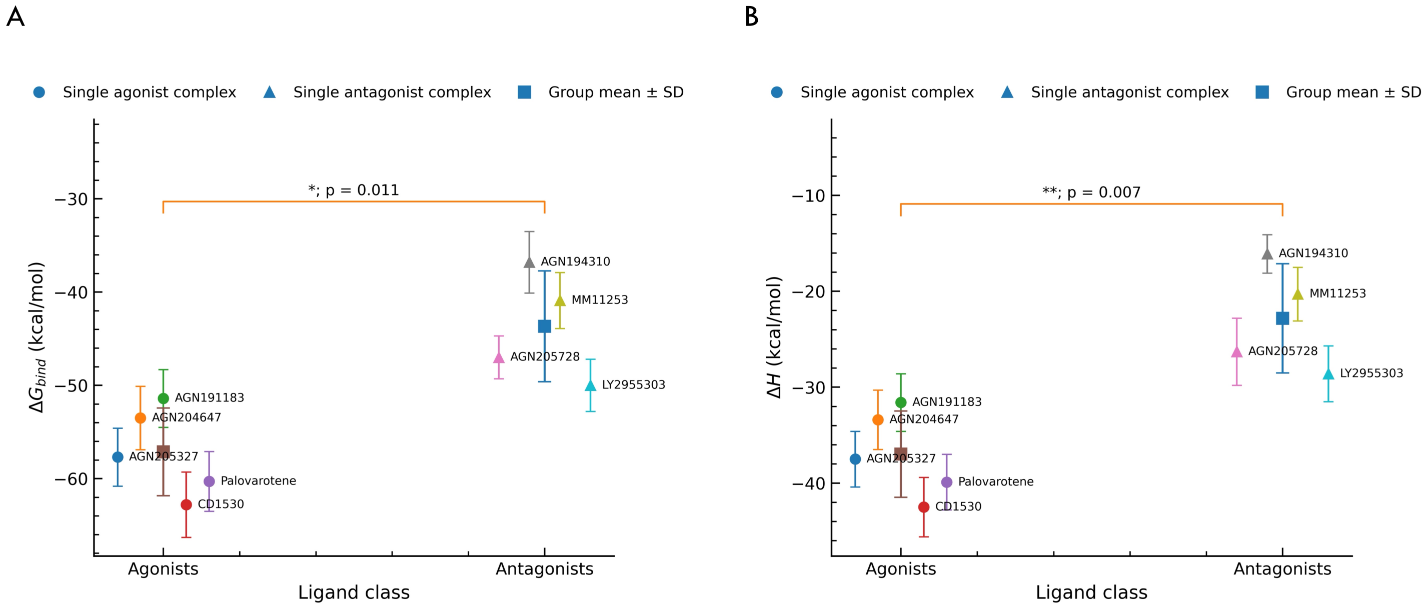

Figure S10. Receptor-specific comparison of MM-PBSA-derived (A) binding free energies G_{bind} and (B) enthalpic contributions ΔH_{eff} for hRARγ agonist and antagonist complexes**.** Individual points represent mean values for single receptor–ligand complexes, labeled by ligand name. Squares and error bars indicate group mean ± SD. The bracket indicates the result of the group-level statistical comparison.

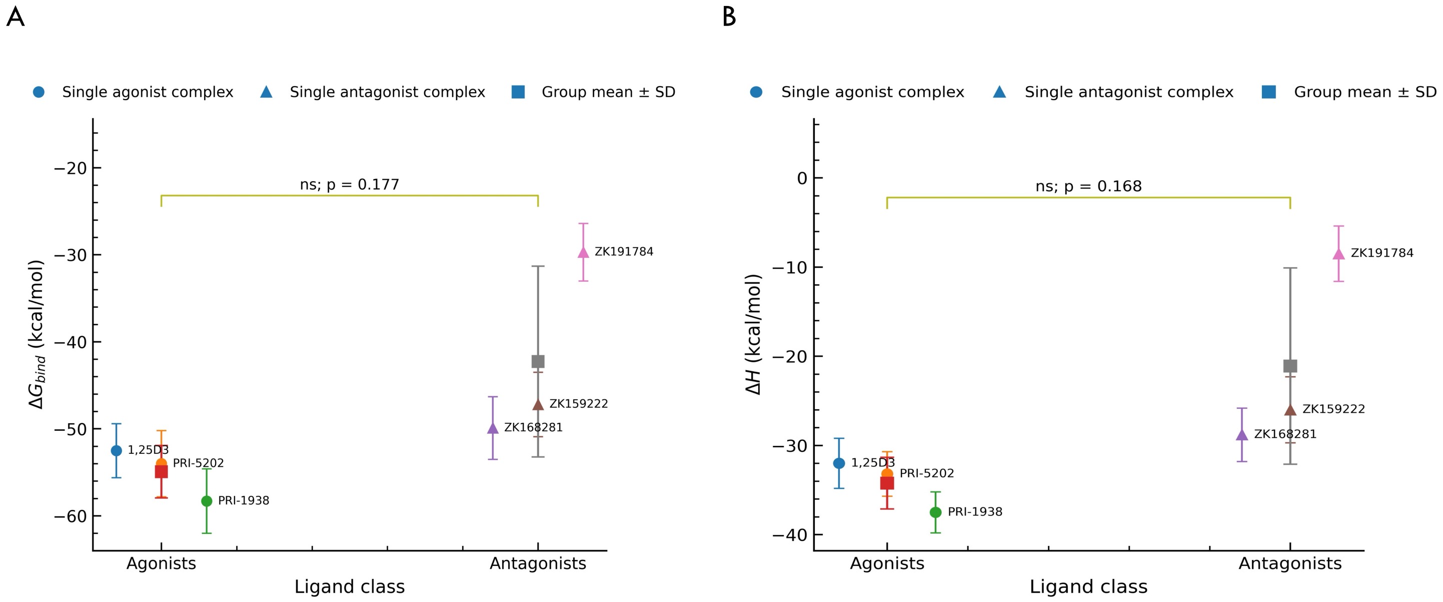

Figure S11. Receptor-specific comparison of MM-PBSA-derived (A) binding free energies G_{bind} and (B) enthalpic contributions ΔH_{eff} for *h*VDR agonist and antagonist complexes**.** Individual points represent mean values for single receptor-ligand complexes, labeled by ligand name. Squares and error bars indicate group mean ± SD. The bracket indicates the result of the group-level statistical comparison.
